# Factors associated with workaholism in nurses’ mental health: integrative review

**DOI:** 10.1590/1518-8345.7046.4218

**Published:** 2024-07-29

**Authors:** Nanielle Silva Barbosa, Jefferson Abraão Caetano Lira, Amanda Alves de Alencar Ribeiro, Eukália Pereira da Rocha, Maria José Quina Galdino, Márcia Astrês Fernandes

**Affiliations:** 1 Universidade Federal do Piauí, Departamento de Pós-Graduação em Enfermagem, Teresina, PI, Brazil.; 2 Scholarship holder at the Coordenação de Aperfeiçoamento de Pessoal de Nível Superior (CAPES), Brazil.; 3 Universidade Estadual do Norte do Paraná, Departamento de Enfermagem, Bandeirantes, PR, Brazil.; 4 Scholarship holder at the Conselho Nacional de Desenvolvimento Científico e Tecnológico (CNPq), Brazil.

**Keywords:** Nurses, Addictive Behavior Addictive, Work, Working Conditions, Mental Health, Occupational Health

## Abstract

**Objective:**

to synthesize the main scientific evidence available on the factors associated with workaholism in nurses’ mental health.

**Method:**

this is an integrative review carried out in seven databases. The sample consisted of 11 studies. The Level of Evidence classification followed the model described by Melnyk and Fineout-Overholt. Methodological quality was assessed using the Checklist for Analytical Cross-Sectional Studies. Data analysis and synthesis were carried out in a qualitative and descriptive manner, respectively.

**Results:**

the factors associated with workaholism were burnout, stress, anxiety, depression, sleep-related problems, low ability to concentrate and negative incidents at work, which affected the mental health of nurses.

**Conclusion:**

the synthesis revealed that workaholism was related to perceived stress at work, emotional exhaustion, depersonalization and anxious and depressive symptoms, which resulted in low professional effectiveness and poor sleep quality among workaholic professionals.

## Highlights

**(1)** Stress and professional exhaustion were related to workaholism.

**(2)** Symptoms of anxiety and depression can be triggered due to workaholism.

**(3)** Workaholism contributes to sleep problems and negative incidents at work.

**(4)** Workaholism interferes with the quality of nursing care.

## Introduction

 Workaholism is an American expression used to designate an addiction to work, also known as work addiction, referring to the individual’s psychopathological dependence on their work activities. This is also among the main causes of physical and mental illness in workers. This phenomenon is usually progressively developed and manifests itself through work behaviors that affect different aspects, mainly related to social, occupational and health demands ^(^ ^1^ ^)^ . 

 When characterizing the state of work addiction, two important assessment dimensions are delimited: excessive and compulsive work. Excessive work is associated with the behavioral dimension and refers to the addictive behavior of working excessively for an extended period of hours, in addition to having difficulty detaching from work during moments of rest or vacation and getting involved in multiple simultaneous projects. Compulsive work is related to the cognitive dimension and is associated with internal cognitive pressure, beliefs and thoughts related to work that lead to compulsive behaviors and attitudes ^(^ ^2^ ^)^ . 

 Workaholism is also associated with pleasure and job satisfaction. However, this correlation has been questioned and modified as a result of mental illness and somatic manifestations caused by workaholism ^(^ ^3^ ^)^ . Other work-related behavioral nuances can be confused with workaholism. Engagement at work, for example, is also associated with excessive working hours and extensive involvement in work activities. However, unlike addiction, engagement is linked to satisfactory performance at work together with feelings of empowerment, positive affect and quality of health ^(^ ^4^ ^)^ . 

 It is understood that the circumstances and motivations associated with workaholism belong to a complexity of multidimensional factors, which may include: oscillation or lack of self-esteem, feeling of inferiority, fear of failure, desire for achievement, accentuated organizational demands and social pressure, due to the constant appreciation of high productivity and performance ^(^ ^5^ ^)^ . 

 Workaholics, when performing their duties excessively and compulsively, give up moments of rest, leisure and/or social interactions with spouses, family and friends. However, despite their intense dedication to work, they generally are not able to achieve the desired performance due to the increased vulnerability to work incapacity and the impacts on biopsychosocial health caused by workaholism ^(^ ^6^ ^)^ . 

 Although this phenomenon has a wide prevalence in different professional categories, such as 42.1% ^(^ ^7^ ^)^ among engineers, 44.9% ^(^ ^8^ ^)^ among doctors and 58.3% ^(^ ^9^ ^)^ among sports coaches, there is a marked tropism between workaholism and nurses ^(^ ^10^ ^-^ ^11^ ^)^ . These workers are exposed to situations and events that can lead to dysfunctional behaviors and increase the risks of dependence at work. 

 Nurses deal daily with demands that involve closeness to patients and families, requiring a mastery of interpersonal skills, empathy and compassion. The demands for the effective development of these skills, together with the high workload and demand of work (more than 40 hours per week), can trigger negative repercussions on the health of these professionals, with an increase in psychological exhaustion (Burnout), secondary traumatic stress and workaholism ^(^ ^12^ ^)^ . 

 Addictive behaviors among nurses and their associated factors allow an explanatory outline of the dimension of this problem. Such indications and considerations reveal the significant relationship between workaholism and changes in the physical and psychological well-being of nurses ^(^ ^1^ ^,^ ^13^ ^-^ ^14^ ^)^ . 

 Prevalence studies estimate average rates between 13.77% ^(^ ^15^ ^)^ and 37% ^(^ ^16^ ^)^ of workaholic nurses. Workaholic nurses are associated with physical and mental health problems, such as difficulties sleeping and/or staying awake during work, mild to moderate depression and negative implications for social and family interaction and the quality of care provided to patients ^(^ ^12^ ^,^ ^15^ ^,^ ^17^ ^)^ . 

It is noted that addiction to work is associated with multidimensional factors. Evidence about workaholism among nurses is important to guide the promotion of occupational health, with the aim of improving mental health, quality of life, satisfaction and performance at work and, consequently, the quality of nursing care provided, in addition to identifying possible gaps to be investigated on the topic.

A critical and detailed analysis of the factors associated with workaholism is essential for expanding understanding of the aspects inherent to work addiction. Furthermore, this evidence may contribute to guiding health managers in planning preventive strategies aimed at building protective behaviors for workers’ mental health in work contexts.

Based on the above, this study aims to synthesize the main scientific evidence available on the factors associated with workaholism in the mental health of nurses.

## Methods

### Study design

 This is an integrative review of the literature, in which the following steps were covered: elaboration of the guiding question, search and selection of primary studies, evaluation of primary studies, data analysis and presentation of the review ^(^ ^18^ ^)^ . The writing of the study followed the recommendations of the Preferred Reporting Items for Systematic Reviews and Meta-Analyses (PRISMA) ^(^ ^19^ ^)^ . 

 The protocol for this review was registered with the Open Science Framework (OSF), and is available for access through the link: https://osf.io/r9pnw/ , presenting the DOI identifier: 10.17605 /OSF.IO/R9PNW ^(^ ^20^ ^)^ . 

### Period

 The study was carried out from March 1 ^st^ to July 31 ^st^ , 2023. 

### Guiding question

 The guiding question defined to conduct this integrative review was: “what is the scientific evidence about the factors associated with workaholism in nurses’ mental health?” To elaborate this question, the acronym PICo (Population, Interest and Context) was adopted ^(^ ^21^ ^)^ , with P=population (nurses), I=interest (factors associated with workaholism in mental health) and Co=context (work). 

### Eligibility criteria

Inclusion criteria were considered: primary studies related to the theme, carried out with nurses, without time or language delimitation. The exclusion criteria were: course completion works, dissertations, theses, editorials and those that did not answer the guiding question.

### Search and selection of studies

 The search for primary studies took place on April 5 ^th^ , 2023, in the online Medical Literature Analysis and Retrieval System (MEDLINE) databases via PubMed, Cumulative Index to Nursing and Allied Health Literature (CINAHL-Ebsco), Web of Science Core Collection, Scopus, Embase, Nursing Database (BDENF) and Latin American and Caribbean Literature in Health Sciences (LILACS), via the Virtual Health Library (VHL). The databases were accessed free of charge through the Periodicals Portal of the Coordination for the Improvement of Higher Education Personnel (CAPES). The selection of primary studies was carried out between April 6 ^th^ and May 20 ^th^ , 2023. 

Initially, a preliminary survey was carried out on the topic with the purpose of identifying the main terms in Portuguese and English used as descriptors and keywords in studies. Then, the descriptors and keywords were established, according to the PICo acronym and according to the specificities of the databases.

 The search terms selected in Medical Subject Headings (MeSH) were applied to MEDLINE, Web of Science Core Collection and Scopus, the CINAHL Subject Headings to CINAHL and the Emtree Terms to Embase. The terms in Portuguese, Spanish and English, selected from the Health Sciences Descriptors (DeCS), were used in the BDENF and LILACS databases. The search strategies were developed through the combination of descriptors and keywords, using the Boolean operators OR and AND, according to Figure 1 . 

**Figure 1:** - Search strategies in the databases consulted to carry out the integrative review. Teresina, PI, Brazil, 2023

**Databases**	**Search strategy**
MEDLINE, via PubMed	((((“nurses”[MeSH Terms]) OR (“nurse”[All Fields])) OR (“nursing personnel”[All Fields])) AND (((“workaholism”[All Fields]) OR (“workaholic”[All Fields])) OR (“work addiction”[All Fields]))) AND ((“work”[MeSH Terms]) OR (“work”[All Fields]))
CINAHL	( (MH “Nurses”) OR “Nurses” OR “nurse” OR ““Nursing Personnel”” ) AND ( “workaholism” OR “workaholic” OR ““work addiction”” ) AND ( (MH “Work”) OR “work” )
Web of Science Core Collection	(ALL=(nurses) OR ALL=(nurse) OR ALL=(“nursing personnel”)) AND (ALL=(workaholism ) OR ALL=(workaholic ) OR ALL=(“work addiction”)) AND (ALL=(work))
Scopus	(( TITLE-ABS-KEY ( nurse ) OR TITLE-ABS-KEY ( nurses ) OR TITLE-ABS-KEY ( “nursing personnel” ) )) AND (( TITLE-ABS-KEY ( workaholism ) OR TITLE-ABS-KEY ( workaholic ) OR TITLE-ABS-KEY ( “work addiction” ) )) AND (TITLE-ABS-KEY ( work ))
Embase	(‘nurse’/exp OR nurse OR ‘community health nurse’ OR ‘community health nurses’ OR ‘nurse’ OR ‘nurse, community health’ OR ‘nurses’ OR ‘nurses, community health’ OR ‘nurses, public health’ OR ‘public health nurse’ OR ‘public health nurses’) AND (‘workaholism’/exp OR workaholism OR ‘work addiction’ OR ‘workaholic behavior’ OR ‘workaholism’) AND (‘work’/exp OR work OR ‘job’ OR ‘job description’ OR ‘work’)
BDENF e LILACS, via BVS	((mh:(Enfermeiras e Enfermeiros)) OR (Enfermeira) OR (Enfermeira e Enfermeiro) OR (Enfermeiras) OR (Enfermeiro e Enfermeira) OR (Enfermeiros e Enfermeiras)) AND ((mh:(“Comportamento aditivo”)) OR (“Conduta aditiva”)) AND ((mh:(Trabalho)) OR (Trabalho)) AND ( db:(“LILACS” OR “BDENF”)) ((mh:(“Enfermeras y Enfermeros”)) OR (Enfermeras ) OR (Enfermeros)) AND ((mh:(“Conducta Adictiva”)) OR (“Conducta Adictiva”)) AND ((mh:(Trabajo)) OR (Trabajo)) AND ( db:(“LILACS” OR “BDENF”)) ((mh:(nurses)) OR (nurses)) AND ((mh:(“Behavior, Addictive”)) OR (“Behavior, Addictive”)) AND ((mh:(work)) OR (work)) AND ( db:(“LILACS” OR “BDENF”))

It is noteworthy that the descriptors and keywords referring to Mental Health and Mental Disorder, as well as their English counterparts, used to address the phenomenon of interest, limited the searches. Therefore, no publications were returned in the databases. As a result, these terms were not elements of the search strategies.

 The results identified in the databases were exported to the online software Rayyan ^(^ ^22^ ^)^ , which helped in the detection and exclusion of duplicates and in the selection of studies included in the review. The study selection stage was carried out by two reviewers, independently, in two stages and followed the recommendations (identification, screening and inclusion) of the PRISMA flowchart ^(^ ^19^ ^)^ . Therefore, in the first stage, the titles and abstracts were read and the eligibility criteria were applied. Afterwards, the reviewers met to discuss the disparities in the selection and reach a consensus. In the next stage, the texts were read in full and the eligibility criteria were applied again. Situations of disagreement, at the end of this stage, were resolved with the opinion of a third reviewer. 

It should be noted that the manual search in the reference list of included primary studies was carried out with the purpose of identifying additional evidence related to the topic of interest.

### Data collection

 Data collection corresponding to the characterization of the studies occurred using an adapted data extraction form ^(^ ^23^ ^)^ , with the following variables being extracted: title, authorship, year of publication, country, periodical, objective of the study, study design , main results, level of evidence and methodological quality. 

This stage was carried out by two reviewers, independently, in May 2023. In cases where disagreements occurred, a meeting was held for discussion until a consensus was reached.

### Data processing and analysis

 Data analysis and synthesis were carried out in a qualitative and descriptive manner, respectively. To classify the level of evidence (LE) of studies, the model proposed by Melnyk and Fineout-Overholt ^(^ ^24^ ^)^ was used, which is divided into the following levels: level I – Evidence from a systematic review or meta-analysis of all relevant randomized controlled clinical trials or originating from clinical guidelines based on systematic reviews of randomized controlled clinical trials; level II – Evidence derived from at least one well-designed randomized controlled clinical trial; level III – Evidence obtained from well-designed clinical trials without randomization; level IV – Evidence from well-designed cohort and case-control studies; level V – Evidence originating from a systematic review of descriptive and qualitative studies; level VI – Evidence derived from a single descriptive or qualitative study; and level VII – Evidence arising from the opinion of authorities and/or reports from expert committees. The Checklist for Analytical Cross-Sectional Studies was used to evaluate the methodological quality of the publications. This tool consists of eight questions. There is no minimum score to determine whether a research has quality or not, however, the more “yes (S)” responses, the better the methodological quality identified ^(^ ^25^ ^)^ . 

The information obtained was presented through tables, in which the studies are characterized according to the variables of interest collected.

## Results

**Figure 2:**
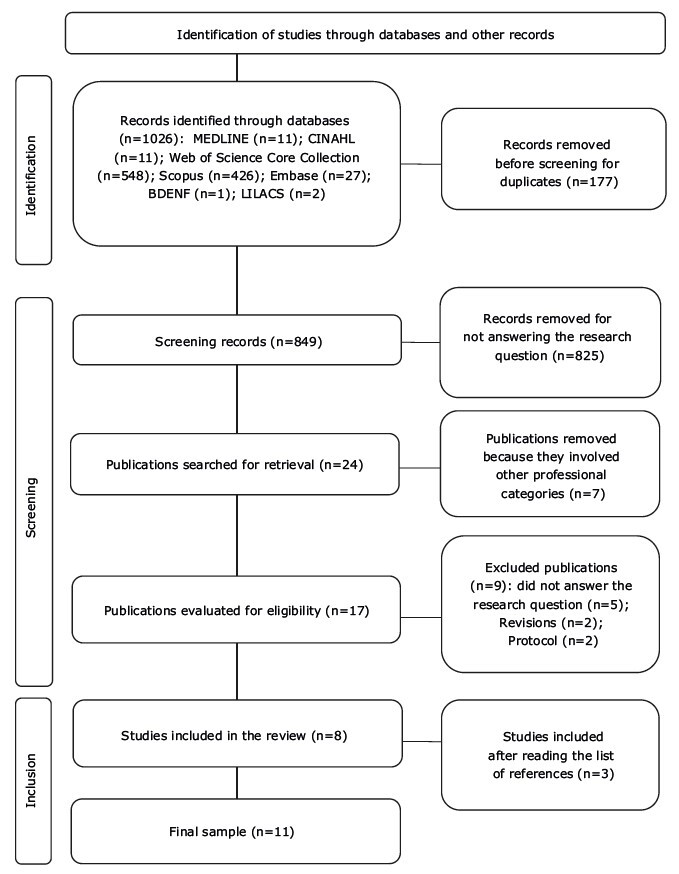
- Flowchart of selection of studies included in the integrative review. Teresina, PI, Brazil, 2023

 The search in the databases returned 1026 publications, 177 of which were removed due to duplicates. Thus, 849 studies were subjected to title and abstract reading. After applying the eligibility criteria, 825 studies were excluded for not answering the guiding question and seven studies for involving other professional categories, leaving 17 studies that were read in full. After this stage, five studies were excluded for not answering the guiding question, two studies for being literature reviews and two for being protocols. Therefore, eight primary studies were selected to compose the sample. Reading the reference list allowed the inclusion of three more articles, totaling 11 studies. In Figure 2 , you can find the flowchart of the selection stage of the primary studies included in this review. 

 The descriptive synthesis of primary studies according to authorship, year of publication, country of study, journal, type of study, sample, setting and level of evidence is presented in Figure 3 . 

**Figure 3:** - Summary of studies included in the integrative review. Teresina, PI, Brazil, 2023

**Authorship and year**	**Country**	**Journal**	**Study design, sample and setting**	**LE** ^*^
Ariapooran (2019) ^(^ ^15^ ^)^	Iran	Iranian Journal of Nursing and Midwifery Research	Cross-sectional and correlational analytical study Sample: n= 247 Setting: three hospitals Industry: not specified	VI
Adolfo, et al. (2021) ^(^ ^16^ ^)^	Saudi Arabia	Perspective in Psychiatric Care	Cross-sectional study Sample: n= 427 Setting: four tertiary public hospitals Sector of activity: emergency, outpatient clinic, medical clinic, surgical clinic, nephrology, obstetric center, intensive care unit and administration	VI
Kubota, et al. (2010) ^(^ ^26^ ^)^	Japan	Industrial Health	Cross-sectional study Sample: n= 312 Setting: two university hospitals Sector of activity: medical clinic, surgical clinic and emergency	VI
Jenaabadi, et al. (2017) ^(^ ^27^ ^)^	Iran	Journal of Health Promotion Management	Descriptive and correlational study Sample: n= 350 Setting: university hospitals Industry: not specified	VI
Nonnis, et al. (2018) ^(^ ^28^ ^)^	Italy	Open Psychology Journal	Cross-sectional study Sample: n= 614 Setting: six hospitals Sector of activity: medical clinic, pediatrics, oncology, psychiatry, obstetrics, neurology, radiotherapy and emergency	VI
Andreassen, et al. (2018) ^(^ ^29^ ^)^	Norway	Industrial Health	Cross-sectional study Sample: n= 1781 Scenario: online Industry: not specified	VI
Kwak, et al. (2018) ^(^ ^30^ ^)^	Korea	Journal of Addictions Nursing	Cross-sectional study Sample: n= 278 Setting: three university hospitals Area of activity: intensive care unit, surgical center and emergency	VI
Almeida, et al. (2020) ^(^ ^31^ ^)^	Brazil	*Revista Latino-Americana de Enfermagem*	Cross-sectional study Sample: n= 333 Scenario: 47 Brazilian public universities Industry of activity: Postgraduate Nursing Programs	VI
Galdino, et al. (2021) ^(^ ^32^ ^)^	Brazil	*Acta Paulista de Enfermagem*	Cross-sectional study Sample: n= 368 Scenario: 47 Brazilian public universities Industry of activity: Postgraduate Nursing Programs	VI
Borges, et al. (2021) ^(^ ^33^ ^)^	Portugal and Spain	Journal of Nursing Management	Multicenter, comparative and cross-sectional study Sample: n= 333 Setting: hospital environment Industry: not specified	VI
Ruíz-Garcia, et al. (2022) ^(^ ^34^ ^)^	Spain	Intensive & Critical Care Nursing	Cross-sectional, descriptive, quantitative and correlational study Sample: n= 219 Setting: hospital environment Area of activity: intensive care unit, cardiac intensive care unit and emergency	VI

* LE = Level of evidence

 The years 2018 and 2021, together, concentrated six productions ^(^ ^16^ ^,^ ^28^ ^-^ ^30^ ^,^ ^32^ ^-^ ^33^ ^)^ . The studies were carried out in countries in Asia ^(^ ^15^ ^-^ ^16^ ^,^ ^26^ ^-^ ^27^ ^,^ ^30^ ^)^ , Europe ^(^ ^28^ ^-^ ^29^ ^,^ ^33^ ^-^ ^34^ ^)^ and South America ^(^ ^31^ ^-^ ^32^ ^)^ , two of which are published in the journal Industrial Health ^(^ ^26^ ^,^ ^29^ ^)^ . Regarding the methodological approach, the studies were characterized as cross-sectional ^(^ ^15^ ^-^ ^16^ ^,^ ^26^ ^-^ ^34^ ^)^ , with a sample of participants between 1781 ^(^ ^29^ ^)^ and 219 ^(^ ^34^ ^)^ nurses, being carried out in a hospital setting ^(15-16,26 -28,30,33-34)^ , online ^(^ ^29^ ^)^ and in universities ^(^ ^31^ ^-^ ^32^ ^)^ and classified with level of evidence VI ^(^ ^15^ ^-^ ^16^ ^,^ ^26^ ^-^ ^34^ ^)^ . Figure 4 shows the objectives and main information about the results of the included studies. 

**Figure 4:** - Summary of studies included in the integrative review according to objectives and main results. Teresina, PI, Brazil, 2023

**Primary study**	**Objective**	**Main results**
Ariapooran (2019) ^(^ ^15^ ^)^	To investigate the role of workaholism in predicting sleep problems and depression among Iranian nurses.	Workaholism was positively correlated with problems associated with sleep and depression
Adolfo, et al. (2021) ^(^ ^16^ ^)^	Determine whether nurses’ workaholic tendencies and demographic variables predict quality of professional life.	Workaholic tendencies were predictive factors of burnout and Secondary Traumatic Stress.
Kubota, et al. (2010) ^(^ ^26^ ^)^	To examine the association between workaholism, tendency to compulsively overwork, and sleep problems among Japanese nurses.	Associations have been identified between workaholism and problems related to sleep quality.
Jenaabadi, et al. (2017) ^(^ ^27^ ^)^	To determine the correlation of workaholism with work stress and professional burnout in nurses.	There was a significant and positive correlation between workaholism and work-related stress and professional burnout.
Nonnis, et al. (2018) ^(^ ^28^ ^)^	Contribute to ongoing research on the relationship between workaholism and burnout among nurses.	Workaholism has been identified as a predictor of burnout.
Andreassen, et al. (2018) ^(^ ^29^ ^)^	Investigate working conditions, sleep and health.	Workaholism was identified as a consistent predictor of negative work-related incidents, particularly involuntary naps at work, which almost caused damage to patients and equipment.
Kwak, et al. (2018) ^(^ ^30^ ^)^	Outline the relationships between work dependence and quality of professional life among nurses in university hospitals.	Workaholism has been identified as an influential factor in burnout and secondary traumatic stress.
Almeida, et al. (2020) ^(^ ^31^ ^)^	To identify the prevalence and factors associated with workaholism among stricto sensu postgraduate nursing professors.	Factors such as poor sleep, low concentration capacity and work anxiety were associated with the dimensions of workaholism.
Galdino, et al. (2021) ^(^ ^32^ ^)^	Verify the association of burnout with workaholism and quality of life among master’s and/or doctorate nursing professors.	Being identified as a workaholic worker significantly increased the chances of high levels of emotional exhaustion, depersonalization and low professional effectiveness.
Borges, et al. (2021) ^(^ ^33^ ^)^	Identify and compare the levels of workaholism, engagement and family interaction between Portuguese and Spanish nurses.	Nurses with stress scored higher average levels for workaholism and excessive work.
Ruíz-Garcia, et al. (2022) ^(^ ^34^ ^)^	Investigate the prevalence of workaholism, as well as the relationship between work-family interaction in emergency and intensive care nurses.	Perceived stress at work has been related to workaholism.

 Regarding the objectives of the studies, there was an interest in examining the association between workaholism and sleep-related problems ^(^ ^15^ ^,^ ^26^ ^)^ , outlining the relationship between workaholism, symptoms of anxiety, depression and quality of professional life ^(^ ^16^ ^,^ ^31^ ^)^ , correlate it with stress and professional exhaustion ^(^ ^27^ ^-^ ^28^ ^,^ ^30^ ^,^ ^32^ ^)^ , identify the prevalence and factors associated with workaholism and compare the levels of workaholism, engagement and family interaction ^(^ ^29^ ^,^ ^33^ ^-^ ^34^ ^)^ . The main results indicate that the factors related to workaholism in the mental health of nurses were depression ^(^ ^15^ ^)^ , burnout ^(^ ^16^ ^,^ ^27^ ^-^ ^28^ ^,^ ^30^ ^,^ ^33^ ^-^ ^34^ ^)^ , stress ^(^ ^16^ ^,^ ^27^ ^,^ ^30^ ^,^ ^33^ ^)^ , anxiety ^(^ ^31^ ^)^ , problems related to sleep ^(^ ^15^ ^,^ ^26^ ^,^ ^29^ ^,^ ^31^ ^)^ , low ability to concentrate ^(^ ^15^ ^,^ ^26^ ^,^ ^29^ ^,^ ^31^ ^)^ and negative incidents at work ^(^ ^29^ ^)^ . 

 In the assessment of methodological quality, using the Checklist for Analytical Cross-Sectional Studies, two surveys ^(^ ^27^ ^,^ ^29^ ^)^ received “yes” in the eight items that make up the tool, one survey ^(^ ^28^ ^)^ received “yes” in seven items and eight studies received “yes” in six items ^(^ ^15^ ^-^ ^16^ ^,^ ^26^ ^,^ ^30^ ^-^ ^34^ ^)^ , as shown in Figure 5 . 

**Figure 5:** - Methodological quality of the studies included in the integrative review. Teresina, PI, Brazil, 2023

**Primary Study**	**Q1**	**Q2**	**Q3**	**Q4**	**Q5**	**Q6**	**Q7**	**Q8**
Ariapooran (2019) ^(^ ^15^ ^)^	Yes	Yes	Yes	Yes	No	No	Yes	Yes
Adolfo, et al. (2021) ^(^ ^16^ ^)^	Yes	Yes	Yes	Yes	No	No	Yes	Yes
Kubota, et al. (2010) ^(^ ^26^ ^)^	No	Yes	Yes	Yes	Yes	No	Yes	Yes
Jenaabadi, et al. (2017) ^(^ ^27^ ^)^	Yes	Yes	Yes	Yes	Yes	Yes	Yes	Yes
Nonnis, et al. (2018) ^(^ ^28^ ^)^	No	Yes	Yes	Yes	Yes	Yes	Yes	Yes
Andreassen, et al. (2018) ^(^ ^29^ ^)^	Yes	Yes	Yes	Yes	Yes	Yes	Yes	Yes
Kwak, et al. (2018) ^(^ ^30^ ^)^	Yes	Yes	Yes	Yes	No	No	Yes	Yes
Almeida, et al. (2020) ^(^ ^31^ ^)^	Yes	Yes	Yes	Yes	No	No	Yes	Yes
Galdino, et al. (2021) ^(^ ^32^ ^)^	Yes	Yes	Yes	Yes	No	No	Yes	Yes
Borges, et al. (2021) ^(^ ^33^ ^)^	Yes	Yes	Yes	Yes	No	No	Yes	Yes
Ruíz-Garcia, et al. (2022) ^(^ ^34^ ^)^	Yes	Yes	Yes	Yes	No	No	Yes	Yes

Q1= Were the sample inclusion criteria clearly defined? Q2= Are the study subjects and setting described in detail? Q3= Was exposure measured validly and reliably? Q4= Were objective and standardized criteria used to measure the condition? Q5= Were confounding factors identified? Q6= Have strategies for dealing with confounding factors been stated? Q7= Were the results measured in a valid and reliable way? Q8= Was appropriate statistical analysis used?

## Discussion

 Nurses constantly deal with organizational and personal demands, physical, mental and emotional health and are more vulnerable to the development of addictive behaviors associated with work, the consequences of which result in psychological suffering and can directly impact the quality of the work performed ^(^ ^1^ ^,^ ^35^ ^)^ . 

 Among the studies identified, the majority were developed with nurses working in the hospital setting. It was pointed out that there is a relationship between workaholism and professional burnout ^(^ ^27^ ^-^ ^28^ ^,^ ^30^ ^)^ . However, establishing a connection between the two is complex, as this relationship is multifaceted, that is, in addition to psychological factors, it involves the perspective of personality, the individual’s behavior and their social relationship with the forms of work organization ^(^ ^16^ ^)^ . 

 The presence of workaholism and burnout among health professionals can be related to the characteristics of medical practice, which does not admit errors or failures in care. In order to avoid errors in the care process, nurses tend to work hard and be overloaded with tasks ^(^ ^36^ ^-^ ^37^ ^)^ . 

 In this context, people addicted to work have a strong intrinsic motivation to work, which they cannot resist. This motivation may be related to the search for satisfaction and happiness at work, improving the financial situation, characteristics of the organizational environment, pressure imposed by superiors, job promotion or even as an alternative to escape family conflicts ^(^ ^38^ ^)^ . 

 It is clear that excessive work, one of the dimensions of workaholism, causes harm to psychophysical and vocational well-being. The little time spent resting leads to the worker’s cognitive and emotional exhaustion, contributing to low occupational performance, interference in interactions with other people, dissatisfaction with work, chronic fatigue, aggressive behaviors, irritability, negative thoughts, frustration and hopelessness. Consequently, there is an increase in absenteeism and turnover among these professionals ^(^ ^30^ ^,^ ^38^ ^)^ . 

 Exhaustion can be triggered by situations of chronic exposure to stressors, with occupational stress being another factor that can be related to the components of workaholism ^(^ ^39^ ^)^ . Nursing is a professional category characterized by assuming multiple responsibilities, which makes nurses prone to excessive work, in addition to being exposed to the risk of workaholism and an environment that contributes to the development of perceived stress and secondary traumatic stress ^(^ ^40^ ^-^ ^42^ ^)^ . 

 Reinforcing the findings on the relationship between workaholism and occupational stress, it is observed that nurses with tendencies towards workaholism present higher levels of secondary traumatic stress ^(^ ^27^ ^,^ ^30^ ^,^ ^33^ ^-^ ^34^ ^)^ . A plausible explanation for this relationship is that workaholic workers tend to exhibit characteristics of perfectionism. Perfectionist people have greater difficulty delegating tasks, believing that they are the only ones capable of carrying out a certain activity. These attitudes reflect on involvement in conflicts with co-workers and cause tensions in their interpersonal relationships. As a consequence of these negative interactions, higher levels of stress are identified ^(^ ^2^ ^)^ . 

 The longer a nurse is exposed to negative events at work, the greater the likelihood of negative effects in other areas of their personal life. The few hours that remain in the daily lives of workaholic nurses are insufficient for them to be able to adopt strategies to reduce stress and protect themselves against burnout ^(^ ^43^ ^-^ ^44^ ^)^ . 

 Another point that requires attention is that constant dedication to work, without considering the need for rest for the body and mind, can lead to chronic stress which, in turn, can cause more serious health problems, such as mental disorders, problems related to sleep, excessive consumption of Psychoactive Substances (PAS), cognitive problems, endocrine diseases and cardiovascular problems ^(^ ^45^ ^)^ . 

 Regarding psychological suffering, arising from the development of mental disorders and workaholism, it is clear that the presence of Obsessive-Compulsive Disorder (OCD) and symptoms of anxiety and/or depression can lead to workaholism and *vice versa* ^(^ ^1^ ^,^ ^15^ ^)^ . Research even suggests that workaholics are more anxious than depressed ^(^ ^46^ ^)^ . 

 People with these characteristics have difficulty dealing with occupational stress and may develop mental health problems ^(^ ^47^ ^)^ . It is believed that, when faced with threatening situations, work can act as an escape mechanism related to negative feelings. Another explanation for this relationship could be that anxious people fear failing and/or refusing tasks, while depressed people work more slowly and need to compensate by working longer hours to complete the work. Both attitudes result in excessive work and workload ^(^ ^48^ ^)^ . 

 Furthermore, when considering that workers characterized as workaholics are people who think persistently and frequently about work demands, they often avoid social interaction. By adopting these habits, workers become vulnerable to showing symptoms of negative mood and depression, even when they are not involved in work activities ^(^ ^15^ ^,^ ^49^ ^)^ . 

 In addition to depression, feelings of guilt and anxiety are commonly experienced by workaholics, and one of the items on the workaholism measurement scale states: “I feel guilty when I take time off from work” ^(^ ^50^ ^)^ . Anxiety symptoms are felt because workaholics are generally driven to achieve their goals in a competitive manner. Therefore, time spent on non-work activities can be seen as a period during which they are “prevented” from competing ^(^ ^51^ ^)^ . 

 The presence of these characteristic symptoms of mental suffering and workaholism itself contribute to the development of sleep-related problems. Workers with workaholism have a longer sleep latency period, as excessive work pace is one of the factors that affects sleep stages ^(^ ^31^ ^,^ ^52^ ^)^ . Workers classified as workaholics may also complain of insufficient sleep, difficulty waking up in the morning (DAM), excessive daytime sleepiness (EDS), feeling tired upon waking up and insomnia ^(^ ^15^ ^,^ ^26^ ^)^ . 

 Difficulty and tiredness when waking up in the morning have a greater association with the cognitive component of workaholism. Regarding this component, low psychological detachment from work predicts negative morning activation and fatigue. Thinking persistently and frequently about work, even when not working, can cause autonomic arousal and emotional distress through cognitive activation, which results in greater fatigue, similar to what occurs in insomnia ^(^ ^53^ ^-^ ^54^ ^)^ . 

 These deficits in sleep quality can have an impact on cognitive and physical performance, reducing alertness, mood, attention and memory and may be associated with an increased risk of errors and accidents at work ^(^ ^55^ ^)^ . Furthermore, high task demands cause mental and physical tension, which can result in involuntary naps and increase the risk of errors in patient care and possible accidents at work ^(^ ^29^ ^)^ . 

 Another scenario in which nurses work, investigated by the studies, was the area of teaching in higher education. Among teaching nurses, working compulsively, excessively and being a workaholic increases the chances of high levels of emotional exhaustion, depersonalization and low professional effectiveness, interdependent dimensions of burnout ^(^ ^32^ ^)^ . 

 The duties of a teacher in higher education are related to multiple activities that add up to the restricted time for their execution, which makes the work exhausting. Furthermore, it is identified that the teacher has frequent and persistent thoughts at work, which also characterizes this work as compulsive, as there is a constant demand for productivity aligned with the competitiveness of the academic environment ^(^ ^56^ ^)^ . 

 In addition to exhaustion, work anxiety among teaching nurses was also one of the factors associated with the dimensions of workaholism ^(^ ^31^ ^)^ . The intense pace of work makes the teacher get used to it and is unable to mentally disengage from work activities. This behavior is usually related to feelings of guilt during free time and/or rest, even if this professional realizes that work is affecting him/her negatively ^(^ ^57^ ^)^ . 

 The included studies presented, in the method, a cross-sectional design, classified as level of evidence VI ^(^ ^24^ ^)^ . The type of study adopted converges with the objectives listed in the investigations, as it allows the observation of the variables of interest at a given moment and directly by the researcher, being particularly useful for studying the prevalence of a phenomenon in a given population. Furthermore, cross-sectional studies seek to analyze the relationships between risk factors, determining factors and what are supposed to be their consequences or effects ^(^ ^58^ ^)^ . 

 Despite the low level of evidence, researchers can use tools to improve the quality of the presentation of results from cross-sectional studies, such as the Strengthening the Reporting of Observational studies in Epidemiology (STROBE) ^(^ ^59^ ^)^ initiative. When analyzing the studies, it was observed that the majority sought to carefully follow these recommendations as a way of guaranteeing the methodological quality of the investigation, reflected through the score obtained with the application of the Checklist for Analytical Cross-Sectional Studies ^(^ ^25^ ^)^ . 

Regarding limitations, the level of evidence of the included productions is highlighted, since, as they are cross-sectional studies, it is not possible to make inferences about the cause and effect relationship between workaholism and nurses’ mental health. It is noted that some included studies had weaknesses in their methodological description, such as not specifying the nurse’s sector of activity, insufficient description of data collection and neglect of confounding factors. Despite the identified limitations, we sought to carefully follow the recommendations for the development of integrative reviews.

## Conclusion

Scientific evidence demonstrated that workaholism was related to perceived stress at work, emotional exhaustion, depersonalization and anxious and depressive symptoms, which resulted in low professional effectiveness and poor sleep quality among workaholic professionals.

Workaholism should not only be categorized as a psychological and individual problem, but also a social problem that influences the forms of work organization, the performance of nurses’ technical-scientific activities and therapeutic relationships with the client, as it contributes for workers’ mental illness, absenteeism and sick leave.

It is suggested that new investigations be carried out on the repercussions of workaholism on the mental health of nurses in work settings, such as Primary Health Care, as well as studies that carefully evaluate the cause and effect relationship between workaholism and psycho-emotional symptoms , considering that, as they have a cross-sectional approach, the studies included do not allow establishing a temporal relationship between exposure and effect, which makes it difficult to determine a causal relationship.

The results of this integrative review may contribute to deepening knowledge about the factors associated with workaholism in nurses, addressing the influence of working conditions on occupational and mental health, in addition to providing reflections on the prevention of addictive behaviors at work and encouraging implementation of public policies to promote worker health aimed at nurses and other members of the nursing team.
