# Supplementary material for: Factors associated with workaholism in nurses’ mental health: integrative review
Source: Rev Lat Am Enfermagem. 2024 Jul 29;32:e4218. doi: 10.1590/1518-8345.7046.4218 (PMC11295265; doi:10.1590/1518-8345.7046.4218)
Supplement: Translation

# Translation: Factores asociados al *workaholism* en la salud mental de enfermeros: revisión integrativa

**DOI:** 10.1590/1518-8345.7046.4217

**Authors:** Nanielle Silva Barbosa, Jefferson Abraão Caetano Lira, Amanda Alves de Alencar Ribeiro, Eukália Pereira da Rocha, Maria José Quina Galdino, Márcia Astrês Fernandes

**Affiliations:** 1 Universidade Federal do Piauí, Departamento de Pós-Graduação em Enfermagem, Teresina, PI, Brasil.; 2 Becario (a) de la Coordenação de Aperfeiçoamento de Pessoal de Nível Superior (CAPES), Brasil.; 3 Universidade Estadual do Norte do Paraná, Departamento de Enfermagem, Bandeirantes, PR, Brasil.; 4 Becaria del Conselho Nacional de Desenvolvimento Científico e Tecnológico (CNPq), Brasil.

**Keywords:** Enfermeras y Enfermeros, Conducta Adictiva, Trabajo, Condiciones de Trabajo, Salud Mental, Salud Laboral

## Abstract

**Objetivo:**

sintetizar las principales evidencias científicas disponibles sobre los factores asociados al *workaholism* en la salud mental de enfermeros.

**Método:**

revisión integrativa realizada en siete bases de datos. La muestra estuvo compuesta por 11 estudios. La clasificación del Nivel de Evidencia siguió el modelo descrito por Melnyk y Fineout-Overholt. La calidad metodológica fue evaluada por medio del *Checklist for Analytical Cross-Sectional Studies* . El análisis y síntesis de los datos se realizaron de manera cualitativa y descriptiva, respectivamente.

**Resultados:**

los factores asociados al *workaholism* fueron *burnout* , estrés, ansiedad, depresión, problemas relacionados con el sueño, baja capacidad de concentración e incidentes negativos en el trabajo, los cuales afectaron la salud mental de los enfermeros.

**Conclusión:**

la síntesis reveló que el *workaholism* presentó relación con el estrés percibido en el trabajo, la exhaución emocional, la despersonalización y los síntomas ansiosos y depresivos, lo que resultó en baja eficacia profesional y mala calidad del sueño entre los profesionales adictos al trabajo.

## Destacados

**(1)** El estrés y el agotamiento profesional se relacionaron con el *workaholism* .

**(2)** Los síntomas de ansiedad y depresión pueden ser desencadenados debido al *workaholism* .

**(3)** El *workaholism* contribuye a problemas de sueño e incidentes negativos en el trabajo.

**(4)** El *workaholism* interfiere en la calidad de la asistencia de enfermería.

## Introducción

 El *workaholism* es una expresión americana utilizada para designar el vicio al trabajo, también conocida como adición al trabajo, haciendo referencia a la dependencia psicopatológica del individuo en relación con sus actividades laborales, y está entre las principales causas de enfermedad física y mental en trabajadores. Este fenómeno se desarrolla progresivamente y se manifiesta por intermedio de conductas laborales que alcanzan aspectos diversos, principalmente relacionados con las demandas social, ocupacional y de salud ^(^ ^1^ ^)^ . 

 En la caracterización del estado de adicción al trabajo, se delimitan dos importantes dimensiones de evaluación: trabajo excesivo y compulsivo. El trabajo excesivo está asociado a la dimensión comportamental y se refiere a la conducta adictiva de trabajar excesivamente por un periodo extenso de horas, además de presentar dificultad para desvincularse del trabajo en momentos de descanso o vacaciones e involucrarse en múltiples proyectos simultáneos. El trabajo compulsivo está relacionado con la dimensión cognitiva y se asocia a la presión interna cognitiva, creencias y pensamientos relacionados con el trabajo que llevan a comportamientos y actitudes de compulsión ^(^ ^2^ ^)^ . 

 El *workaholism* también está asociado al placer y a la satisfacción laboral. Sin embargo, esta correlación ha sido cuestionada y modificada debido al deterioro mental y a manifestaciones somáticas provocadas por la adicción al trabajo ^(^ ^3^ ^)^ . Otros matices comportamentales relacionadas con el trabajo pueden confundirse con el *workaholism* . El compromiso laboral, por ejemplo, también se asocia a cargas horarias excesivas y al extenso involucramiento con las actividades laborales. Sin embargo, a diferencia de la adicción, el compromiso se interrelaciona con un desempeño satisfactorio en el trabajo junto con sentimientos de empoderamiento, afecto positivo y calidad en salud ^(^ ^4^ ^)^ . 

 Se entiende que las circunstancias y las motivaciones asociadas al *workaholism* pertenecen a una complejidad de factores multidimensionales, en los cuales pueden incluirse: oscilación o falta de autoestima, sentimiento de inferioridad, miedo al fracaso, deseo de realización, exigencias organizacionales acentuadas y presión social, debido a la valorización constante de alta productividad y desempeño ^(^ ^5^ ^)^ . 

 Los *workaholics* o “adictos al trabajo”, al desempeñar sus funciones de forma excesiva y compulsiva, abdican de momentos de descanso, ocio y/o de interacciones sociales con cónyuges, familiares y amigos. Sin embargo, a pesar de la intensa dedicación al trabajo, generalmente no logran alcanzar el rendimiento deseado debido al aumento de la vulnerabilidad a la incapacidad laboral y a los impactos en la salud biopsicosocial provocados por el *workaholism* ^(^ ^6^ ^)^ . 

 A pesar de que este fenómeno presenta una amplia prevalencia en categorías profesionales diversas, como 42,1% ^(^ ^7^ ^)^ entre ingenieros, 44,9% ^(^ ^8^ ^)^ entre médicos y 58,3% ^(^ ^9^ ^)^ entre entrenadores deportivos, se nota el tropismo acentuado entre *workaholism* y enfermeros ^(^ ^10^ ^-^ ^11^ ^)^ . Estos trabajadores están expuestos a situaciones y eventos que pueden llevar a comportamientos disfuncionales y ampliar los riesgos a la dependencia en el trabajo. 

 Los enfermeros lidian diariamente con demandas que involucran la proximidad con pacientes y familiares, exigiendo un dominio de habilidades interpersonales, empatía y compasión. Las exigencias por el desarrollo efectivo de estas competencias, junto con la elevada carga y demanda de trabajo (superior a 40 horas semanales), pueden desencadenar repercusiones negativas a la salud de estos profesionales, con el aumento del agotamiento psicológico ( *Burnout* ), estrés traumático secundario y *workaholism* ^(^ ^12^ ^)^ . 

 Los comportamientos adictivos entre enfermeros y sus factores asociados permiten un delineamiento explicativo de la dimensión de esta problemática. Tales indicativos y consideraciones revelan la expresiva relación entre el *workaholism* y las alteraciones en el bienestar físico y psicológico del enfermero ^(^ ^1^ ^,^ ^13^ ^-^ ^14^ ^)^ . 

 Estudios de prevalencia estiman tasas medias entre 13,77% ^(^ ^15^ ^)^ y 37% ^(^ ^16^ ^)^ de enfermeros *workaholics* . A los enfermeros *workaholics* , se asocian agravios de salud físicos y mentales, como dificultades para dormir y/o mantenerse despierto durante el trabajo, cuadros de depresión leve a moderada e implicaciones negativas en la interacción social, familiar y en la calidad de la asistencia prestada a los pacientes ^(^ ^12^ ^,^ ^15^ ^,^ ^17^ ^)^ . 

 Se nota que la adicción al trabajo se asocia a factores multidimensionales. Las evidencias acerca del *workaholism* entre los enfermeros son importantes para orientar la promoción de la salud ocupacional, con el fin de mejorar la salud mental, la calidad de vida, la satisfacción y el desempeño en el trabajo y, por consiguiente, la calidad de la asistencia de enfermería prestada, además de identificar posibles lagunas a ser investigadas sobre la temática. 

 El análisis crítico y detallado sobre los factores asociados al *workaholism* es imprescindible para la ampliación del entendimiento sobre los aspectos inherentes a la adicción al trabajo. Además, estas evidencias podrán contribuir para orientar a los gestores en salud en la planificación de estrategias preventivas dirigidas a la construcción de comportamientos protectores a la salud mental de los trabajadores en los contextos laborales. 

 A partir de lo expuesto, este estudio tiene como objetivo sintetizar las principales evidencias científicas disponibles sobre los factores asociados al *workaholism* en la salud mental de enfermeros. 

## Método

### Tipo de estudio

 Se trata de una revisión integrativa de la literatura, en la cual se siguieron las siguientes etapas: elaboración de la pregunta norteadora, búsqueda y selección de los estudios primarios, evaluación de los estudios primarios, análisis de los datos y presentación de la revisión ^(^ ^18^ ^)^ . La redacción del estudio siguió las recomendaciones del *Preferred Reporting Items for Systematic Reviews and Meta-Analyses* (PRISMA) ^(^ ^19^ ^)^ . 

 El protocolo de esta revisión se registró en *Open Science Framework* (OSF), y está disponible para acceso por medio del enlace: https://osf.io/r9pnw/ , presentando el identificador DOI: 10.17605/OSF.IO/R9PNW ^(^ ^20^ ^)^ . 

### Período

El estudio se realizó en el período del 01 de marzo al 31 de julio de 2023.

### Pregunta norteadora

 La pregunta norteadora definida para conducir esta revisión integrativa fue: “¿cuáles son las evidencias científicas acerca de los factores asociados al *workaholism* en la salud mental de enfermeros?”. Para la elaboración de esta pregunta, se adoptó el acrónimo PICo (Población, Interés y Contexto), siendo P=población (enfermeros), I=interés (factores asociados al *workaholism* en la salud mental) y Co=contexto (trabajo). 

### Criterios de elegibilidad

Se consideraron criterios de inclusión: estudios primarios relacionados con la temática, realizados con enfermeros, sin delimitación temporal ni de idioma. Los criterios de exclusión fueron: trabajos de conclusión de curso, disertaciones, tesis, editoriales y aquellos que no respondieran a la pregunta norteadora.

### Búsqueda y selección de los estudios

 La búsqueda de los estudios primarios ocurrió el 05 de abril de 2023, en las bases de datos *Medical Literature Analysis and Retrieval System on-line* (MEDLINE) vía PubMed, *Cumulative Index to Nursing and Allied Health Literature* (CINAHL-Ebsco), *Web of Science Core Collection* , Scopus, Embase, Base de Datos en Enfermería (BDENF) y Literatura Latinoamericana y del Caribe en Ciencias de la Salud (LILACS), vía Biblioteca Virtual en Salud (BVS). Las bases de datos se accedieron gratuitamente mediante el Portal de Periódicos de la *Coordenação de Aperfeiçoamento de Pessoal de Nível Superior* (CAPES). La selección de los estudios primarios se realizó entre el 06 de abril y el 20 de mayo de 2023. 

Inicialmente, se realizó un levantamiento previo sobre el tema con el fin de identificar los principales términos en portugués e inglés utilizados como descriptores y palabras clave en los estudios. A continuación, los descriptores y palabras clave se establecieron, conforme al acrónimo PICo y de acuerdo con las especificidades de las bases de datos.

 Los términos de búsqueda seleccionados en *Medical Subject Headings* (MeSH) se aplicaron en MEDLINE, *Web of Science Core Collection* y Scopus, los CINAHL *Subject Headings* en CINAHL y los *Emtree Terms* en Embase. Los términos en portugués, español e inglés, seleccionados en los Descriptores en Ciencias de la Salud (DeCS), se utilizaron en las bases de datos BDENF y LILACS. Las estrategias de búsqueda se elaboraron mediante la combinación de descriptores y palabras clave, a partir de los operadores booleanos OR y AND, de acuerdo con la Figura 1 . 

**Figura 1:** - Estrategias de búsqueda en las bases de datos consultadas para realizar la revisión integrativa. Teresina, PI, Brasil, 2023

**Base de datos**	**Estrategias de búsqueda**
MEDLINE, via PubMed	((((“nurses”[MeSH Terms]) OR (“nurse”[All Fields])) OR (“nursing personnel”[All Fields])) AND (((“workaholism”[All Fields]) OR (“workaholic”[All Fields])) OR (“work addiction”[All Fields]))) AND ((“work”[MeSH Terms]) OR (“work”[All Fields]))
CINAHL	( (MH “Nurses”) OR “Nurses” OR “nurse” OR “‘’Nursing Personnel’’” ) AND ( “workaholism” OR “workaholic” OR “‘’work addiction’’” ) AND ( (MH “Work”) OR “work” )
*Web of Science Core Collection*	(ALL=(nurses) OR ALL=(nurse) OR ALL=(‘’nursing personnel’‘)) AND (ALL=(workaholism ) OR ALL=(workaholic ) OR ALL=(’‘work addiction’’)) AND (ALL=(work))
Scopus	(( TITLE-ABS-KEY ( nurse ) OR TITLE-ABS-KEY ( nurses ) OR TITLE-ABS-KEY ( ‘’nursing personnel’’ ) )) AND (( TITLE-ABS-KEY ( workaholism ) OR TITLE-ABS-KEY ( workaholic ) OR TITLE-ABS-KEY ( ‘’work addiction’’ ) )) AND (TITLE-ABS-KEY ( work ))
Embase	(‘nurse’/exp OR nurse OR ‘community health nurse’ OR ‘community health nurses’ OR ‘nurse’ OR ‘nurse, community health’ OR ‘nurses’ OR ‘nurses, community health’ OR ‘nurses, public health’ OR ‘public health nurse’ OR ‘public health nurses’) AND (‘workaholism’/exp OR workaholism OR ‘work addiction’ OR ‘workaholic behavior’ OR ‘workaholism’) AND (‘work’/exp OR work OR ‘job’ OR ‘job description’ OR ‘work’)
BDENF y LILACS, via BVS	((mh:(Enfermeiras e Enfermeiros)) OR (Enfermeira) OR (Enfermeira e Enfermeiro) OR (Enfermeiras) OR (Enfermeiro e Enfermeira) OR (Enfermeiros e Enfermeiras)) AND ((mh:(‘’Comportamento aditivo’‘)) OR (’‘Conduta aditiva’’)) AND ((mh:(Trabalho)) OR (Trabalho)) AND ( db:(“LILACS” OR “BDENF”)) ((mh:(“Enfermeras y Enfermeros”)) OR (Enfermeras ) OR (Enfermeros)) AND ((mh:(“Conducta Adictiva”)) OR (“Conducta Adictiva”)) AND ((mh:(Trabajo)) OR (Trabajo)) AND ( db:(“LILACS” OR “BDENF”)) ((mh:(nurses)) OR (nurses)) AND ((mh:(“Behavior, Addictive”)) OR (“Behavior, Addictive”)) AND ((mh:(work)) OR (work)) AND ( db:(“LILACS” OR “BDENF”))

Se destaca que los descriptores y las palabras clave referentes a la Salud Mental y al Trastorno Mental, así como sus correspondientes en inglés, utilizados para atender al fenómeno de interés, limitaron las búsquedas. Por lo tanto, no se obtuvo retorno de publicaciones en las bases de datos. En consecuencia, estos términos no constituyeron elementos de las estrategias de búsqueda.

 Los resultados identificados en las bases de datos fueron exportados para el *software* en línea *Rayyan* ^(^ ^22^ ^)^ , que ayudó en la detección y exclusión de duplicados y en la selección de los estudios incluidos en la revisión. La etapa de selección de los estudios fue realizada por dos revisores, de forma independiente, en dos etapas y siguió las recomendaciones (identificación, cribado e inclusión) del flujo de trabajo PRISMA ^(^ ^19^ ^)^ . De esta manera, en la primera etapa, se realizó la lectura de los títulos y resúmenes y la aplicación de los criterios de elegibilidad. Posteriormente, los revisores se reunieron para discutir las disparidades en la selección y llegar a un consenso. En la etapa siguiente, se llevó a cabo la lectura de los textos completos y se aplicaron, nuevamente, los criterios de elegibilidad. Las situaciones de desacuerdo, al final de esta etapa, se resolvieron con la opinión de un tercer revisor. 

Se resalta que la búsqueda manual en la lista de referencias de los estudios primarios incluidos se realizó con el fin de identificar evidencias adicionales relacionadas con la temática de interés.

### Recolección de datos

 La recolección de los datos correspondiente a la caracterización de los estudios se llevó a cabo mediante el uso de un formulario de extracción de datos adaptado ^(^ ^23^ ^)^ , extrayéndose las siguientes variables: título, autoría, año de publicación, país, periódico, objetivo del estudio, diseño del estudio, principales resultados, nivel de evidencia y calidad metodológica. 

Esta etapa fue realizada por dos revisores, de forma independiente, en el mes de mayo de 2023. En los casos en que ocurrieron divergencias, se realizó una reunión para discusión hasta que se llegó a un consenso.

### Tratamiento y análisis de datos

 El análisis y la síntesis de los datos se realizaron de manera cualitativa y descriptiva, respectivamente. Para la clasificación del nivel de evidencia (NE) de los estudios se utilizó el modelo propuesto por Melnyk y Fineout-Overholt ^(^ ^24^ ^)^ , el cual se divide en los siguientes niveles: nivel I – Evidencias provenientes de revisión sistemática o metanálisis de todos los ensayos clínicos controlados aleatorizados relevantes o provenientes de directrices clínicas basadas en revisiones sistemáticas de ensayos clínicos controlados aleatorizados; nivel II – Evidencias derivadas de, al menos, un ensayo clínico controlado aleatorizado bien delineado; nivel III – Evidencias obtenidas de ensayos clínicos bien delineados sin randomización; nivel IV – Evidencias provenientes de estudios de cohorte y de caso-control bien delineados; nivel V – Evidencias originarias de revisión sistemática de estudios descriptivos y cualitativos; nivel VI – Evidencias derivadas de un único estudio descriptivo o cualitativo; y nivel VII – Evidencias provenientes de la opinión de autoridades y/o informes de comités de expertos. El *Checklist for Analytical Cross-Sectional Studies* se utilizó para evaluar la calidad metodológica de las publicaciones. Esta herramienta consta de ocho preguntas. No hay una puntuación mínima para determinar si una investigación posee calidad o no, sin embargo, cuantas más respuestas “sí (S)”, mejor será la calidad metodológica identificada ^(^ ^25^ ^)^ . 

La información obtenida se presentó mediante cuadros, en los cuales los estudios están caracterizados conforme a las variables de interés recogidas.

## Resultados

 La búsqueda en las bases de datos devolvió 1026 publicaciones, de las cuales 177 fueron eliminadas por duplicados. De este modo, 849 estudios fueron sometidos a la lectura del título y del resumen. Tras la aplicación de los criterios de elegibilidad, 825 estudios fueron excluidos por no responder a la pregunta norteadora y siete estudios por involucrar a otras categorías profesionales, quedando 17 estudios que fueron leídos íntegramente. Después de esta etapa, cinco estudios fueron excluidos por no responder a la pregunta norteadora, dos estudios por ser revisiones de literatura y dos por ser protocolos. Así, ocho estudios primarios fueron seleccionados para componer la muestra. La lectura de la lista de referencias permitió la inclusión de tres artículos más, totalizando 11 estudios. En la Figura 2 , se encuentra el flujograma de la etapa de selección de los estudios primarios incluidos en esta revisión. 

**Figura 2:** - Flujograma de selección de los estudios incluidos en la revisión integrativa. Teresina, PI, Brasil, 2023

 La síntesis descriptiva de los estudios primarios conforme autoría, año de publicación, país del estudio, periódico, tipo de estudio, muestra, escenario y nivel de evidencia se presenta en la Figura 3 . 

**Figura 3:** - Síntesis de los estudios incluidos en la revisión integrativa. Teresina, PI, Brasil, 2023

**Autoría y año**	**País**	**Periódico**	**Tipo de estudio, muestra y escenario**	**NE** ^*^
Ariapooran (2019) ^(^ ^15^ ^)^	Irán	*Iranian Journal of Nursing and Midwifery Research*	Estudio analítico transversal y correlacional Muestra: n= 247 Escenario: tres hospitales Sector de actuación: no especificado	VI
Adolfo, et al. (2021) ^(^ ^16^ ^)^	Arabia Saudita	*Perspective in Psychiatric Care*	Estudio transversal Muestra: n= 427 Escenario: cuatro hospitales públicos terciarios Sector de actuación: emergencia, ambulatorio, clínica médica, clínica quirúrgica, nefrología, centro obstétrico, unidad de terapia intensiva y administración	VI
Kubota, et al. (2010) ^(^ ^26^ ^)^	Japón	*Industrial Health*	Estudio transversal Muestra: n= 312 Escenario: dos hospitales universitarios Sector de actuación: clínica médica, clínica quirúrgica y emergencia	VI
Jenaabadi, et al. (2017) ^(^ ^27^ ^)^	Irán	*Journal of Health Promotion Management*	Estudio descriptivo y correlacional Muestra: n= 350 Escenario: hospitales universitarios Sector de actuación: no especificado	VI
Nonnis, et al. (2018) ^(^ ^28^ ^)^	Italia	*Open Psychology Journal*	Estudio transversal Muestra: n= 614 Escenario: seis hospitales Sector de actuación: clínica general, pediatría, oncología, psiquiatría, obstetricia, neurología, radioterapia y emergencia	VI
Andreassen, et al. (2018) ^(^ ^29^ ^)^	Noruega	*Industrial Health*	Estudio transversal Muestra: n= 1781 Escenario: *online* Sector de actuación: no especificado	VI
Kwak, et al. (2018) ^(^ ^30^ ^)^	Corea	*Journal of Addictions Nursing*	Estudio transversal Muestra: n= 278 Escenario: tres hospitales universitarios Sector de actuación: unidad de terapia intensiva, centro quirúrgico y emergencia	VI
Almeida, et al. (2020) ^(^ ^31^ ^)^	Brasil	*Revista Latino-Americana de Enfermagem*	Estudio transversal Muestra: n= 333 Escenario: 47 universidades públicas brasileñas Sector de actuación: Programas de Posgrado en Enfermería	VI
Galdino, et al. (2021) ^(^ ^32^ ^)^	Brasil	*Acta Paulista de Enfermagem*	Estudio transversal Muestra: n= 368 Escenario: 47 universidades públicas brasileñas Sector de actuación: Programas de Posgrado en Enfermería	VI
Borges, et al. (2021) ^(^ ^33^ ^)^	Portugal y España	*Journal of Nursing Management*	Estudio multicéntrico, comparativo y transversal Muestra: n= 333 Escenario: ambiente hospitalario Sector de actuación: no especificado	VI
Ruíz-Garcia, et al. (2022) ^(^ ^34^ ^)^	España	*Intensive & Critical Care Nursing*	Estudio transversal, descriptivo, cuantitativo y correlacional Muestra: n= 219 Escenario: ambiente hospitalario Sector de actuación: unidad de terapia intensiva, unidad de cuidados intensivos cardíacos y emergencia	VI

* NE = Nivel de evidencia

 Los años 2018 y 2021, juntos, concentraron seis producciones ^(^ ^16^ ^,^ ^28^ ^-^ ^30^ ^,^ ^32^ ^-^ ^33^ ^)^ . Los estudios se desarrollaron en países de Asia ^(^ ^15^ ^-^ ^16^ ^,^ ^26^ ^-^ ^27^ ^,^ ^30^ ^)^ , Europa ^(28-29,33-34^ ) y América del Sur ^(^ ^31^ ^-^ ^32^ ^)^ , de los cuales dos están publicados en el periódico *Industrial Health* ^(^ ^26^ ^,^ ^29^ ^)^ . En relación con el enfoque metodológico, los estudios se caracterizaron como transversales ^(^ ^15^ ^-^ ^16^ ^,^ ^26^ ^-^ ^34^ ^)^ , con una muestra de participantes entre 1781 ^(^ ^29^ ^)^ y 219 ^(^ ^34^ ^)^ enfermeros, siendo realizados en escenario hospitalario ^(^ ^15^ ^-^ ^16^ ^,^ ^26^ ^-^ ^28^ ^,^ ^30^ ^,^ ^33^ ^-^ ^34^ ^)^ , *online* ^(^ ^29^ ^)^ y en universidades ^(^ ^31^ ^-^ ^32^ ^)^ y clasificados con nivel de evidencia VI ^(^ ^15^ ^-^ ^16^ ^,^ ^26^ ^-^ ^34^ ^)^ . La Figura 4 presenta los objetivos y las principales informaciones acerca de los resultados de los estudios incluidos. 

**Figura 4:** - Síntesis de los estudios incluidos en la revisión integrativa según objetivos y principales resultados. Teresina, PI, Brasil, 2023

**Estudio primario**	**Objetivo**	**Principales resultados**
Ariapooran (2019) ^(^ ^15^ ^)^	Investigar el papel de la adicción al trabajo en la predicción de problemas de sueño y depresión entre enfermeros iraníes.	El *workaholism* fue positivamente correlacionado con problemas asociados al sueño y depresión.
Adolfo, et al. (2021) ^(^ ^16^ ^)^	Determinar si las tendencias *workaholic* y las variables demográficas de los enfermeros predicen la calidad de vida profesional.	Las tendencias *workaholic* fueron factores predictores de *burnout* y Estrés Traumático Secundario.
Kubota, et al. (2010) ^(^ ^26^ ^)^	Examinar la asociación entre *workaholism* , la tendencia a trabajar excesivamente de forma compulsiva y problemas de sueño entre enfermeros japoneses.	Se identificaron asociaciones entre la adicción al trabajo y problemas relacionados con la calidad del sueño.
Jenaabadi, et al. (2017) ^(^ ^27^ ^)^	Determinar la correlación de la adicción al trabajo con el estrés laboral y el agotamiento profesional en enfermeros.	Hubo una correlación significativa y positiva entre *workaholism* y estrés relacionado con el trabajo y agotamiento profesional.
Nonnis, et al. (2018) ^(^ ^28^ ^)^	Contribuir a la investigación en curso sobre la relación entre *workaholism* y *burnout* entre enfermeros.	El *workaholism* fue señalado como predictor de *burnout* .
Andreassen, et al. (2018) ^(^ ^29^ ^)^	Investigar sobre condiciones de trabajo, sueño y salud.	La adicción al trabajo fue identificada como un predictor consistente de incidentes negativos relacionados con el trabajo, destacando las siestas involuntarias en el trabajo, lo que casi ocasionó daños a pacientes y equipos.
Kwak, et al. (2018) ^(^ ^30^ ^)^	Delinear las relaciones entre la dependencia del trabajo y calidad de vida profesional entre enfermeros en hospitales universitarios.	La adicción al trabajo fue señalada como un factor influyente en *burnout* y estrés traumático secundario.
Almeida, et al. (2020) ^(^ ^31^ ^)^	Identificar la prevalencia y los factores asociados al *workaholism* entre docentes de posgrado *stricto sensu* en enfermería.	Factores como el sueño insatisfactorio, baja capacidad de concentración y ansiedad laboral estuvieron asociados a las dimensiones del *workaholism* .
Galdino, et al. (2021) ^(^ ^32^ ^)^	Verificar la asociación del *burnout* con *workaholism* y calidad de vida entre docentes de maestría y/o doctorado en enfermería.	Ser identificado como trabajador *workaholic* aumentó significativamente las chances de altos niveles de exhaución emocional, despersonalización y baja eficacia profesional.
Borges, et al. (2021) ^(^ ^33^ ^)^	Identificar y comparar los niveles de *workaholism* , *engagement* e interacción familiar entre enfermeros portugueses y españoles.	Enfermeros con estrés puntuaron niveles medios más altos de adicción al trabajo y trabajo excesivo.
Ruíz-Garcia, et al. (2022) ^(^ ^34^ ^)^	Investigar la prevalencia de la adicción al trabajo, así como la relación entre la interacción trabajo-familia en enfermeros de emergencia y cuidados intensivos.	El estrés percibido en el trabajo fue relacionado con el *workaholism* .

 En cuanto a los objetivos de los estudios, se observó el interés en examinar la asociación entre *workaholism* y problemas relacionados con el sueño ^(^ ^15^ ^,^ ^26^ ^)^ , delinear la relación entre *workaholism* , síntomas de ansiedad, depresión y calidad de vida profesional ^(^ ^16^ ^,^ ^31^ ^)^ , correlacionarlo con estrés y agotamiento profesional ^(^ ^27^ ^-^ ^28^ ^,^ ^30^ ^,^ ^32^ ^)^ , identificar la prevalencia y los factores asociados al *workaholism* y comparar los niveles de *workaholism* , compromiso e interacción familiar ^(^ ^29^ ^,^ ^33^ ^-^ ^34^ ^)^ . Los principales resultados indican que los factores relacionados con el *workaholism* en la salud mental de los enfermeros fueron depresión ^(^ ^15^ ^)^ , *burnout* ^(^ ^16^ ^,^ ^27^ ^-^ ^28^ ^,^ ^30^ ^,^ ^33^ ^-^ ^34^ ^)^ , estrés ^(^ ^16^ ^,^ ^27^ ^,^ ^30^ ^,^ ^33^ ^)^ , ansiedad ^(^ ^31^ ^)^ , problemas relacionados con el sueño ^(^ ^15^ ^,^ ^26^ ^,^ ^29^ ^,^ ^31^ ^)^ , baja capacidad de concentración ^(^ ^15^ ^,^ ^26^ ^,^ ^29^ ^,^ ^31^ ^)^ e incidentes negativos en el trabajo ^(^ ^29^ ^)^ . 

 En la evaluación de la calidad metodológica, por medio del *Checklist for Analytical Cross-Sectional Studies* , dos investigaciones ^(^ ^27^ ^,^ ^29^ ^)^ recibieron “sí” en los ocho ítems que componen la herramienta, una investigación ^(^ ^28^ ^)^ recibió “sí” en siete ítems y ocho estudios recibieron “sí” en seis ítems ^(^ ^15^ ^-^ ^16^ ^,^ ^26^ ^,^ ^30^ ^-^ ^34^ ^)^ , como se presenta en la Figura 5 . 

**Figura 5:** - Calidad metodológica de los estudios incluidos en la revisión integrativa. Teresina, PI, Brasil, 2023

**Estudio primario**	**Q1**	**Q2**	**Q3**	**Q4**	**Q5**	**Q6**	**Q7**	**Q8**
Ariapooran (2019) ^(^ ^15^ ^)^	Sí	Sí	Sí	Sí	No	No	Sí	Sí
Adolfo, et al. (2021) ^(^ ^16^ ^)^	Sí	Sí	Sí	Sí	No	No	Sí	Sí
Kubota, et al. (2010) ^(^ ^26^ ^)^	No	Sí	Sí	Sí	Sí	No	Sí	Sí
Jenaabadi, et al. (2017) ^(^ ^27^ ^)^	Sí	Sí	Sí	Sí	Sí	Sí	Sí	Sí
Nonnis, et al. (2018) ^(^ ^28^ ^)^	No	Sí	Sí	Sí	Sí	Sí	Sí	Sí
Andreassen, et al. (2018) ^(^ ^29^ ^)^	Sí	Sí	Sí	Sí	Sí	Sí	Sí	Sí
Kwak, et al. (2018) ^(^ ^30^ ^)^	Sí	Sí	Sí	Sí	No	No	Sí	Sí
Almeida, et al. (2020) ^(^ ^31^ ^)^	Sí	Sí	Sí	Sí	No	No	Sí	Sí
Galdino, et al. (2021) ^(^ ^32^ ^)^	Sí	Sí	Sí	Sí	No	No	Sí	Sí
Borges, et al. (2021) ^(^ ^33^ ^)^	Sí	Sí	Sí	Sí	No	No	Sí	Sí
Ruíz-Garcia, et al. (2022) ^(^ ^34^ ^)^	Sí	Sí	Sí	Sí	No	No	Sí	Sí

Q1= ¿Se definieron de forma clara los criterios de inclusión en la muestra? Q2= ¿Se describen en detalle los sujetos del estudio y el escenario? Q3= ¿Se midió la exposición de forma válida y fiable? Q4= ¿Se utilizaron criterios objetivos y estandarizados para la medición de la condición? Q5= ¿Se identificaron factores de confusión? Q6= ¿Se declararon estrategias para lidiar con factores de confusión? Q7= ¿Se midieron los resultados de forma válida y fiable? Q8= ¿Se utilizó análisis estadístico apropiado?

## Discusión

 Los enfermeros enfrentan constantemente demandas organizacionales y personales, de salud física, mental y emocional, y presentan una mayor vulnerabilidad al desarrollo de comportamientos adictivos asociados al trabajo, cuyas consecuencias resultan en sufrimiento psicológico y pueden impactar directamente en la calidad del trabajo desempeñado ^(^ ^1^ ^,^ ^35^ ^)^ . 

 Entre los estudios identificados, la mayoría se desarrolló con enfermeros que trabajan en el escenario hospitalario. Se señaló que existe una relación entre la adicción al trabajo y el agotamiento profesional ^(^ ^27^ ^-^ ^28^ ^,^ ^30^ ^)^ . Sin embargo, establecer una conexión entre ambos es complejo, pues esta relación es multifacética, es decir, además de factores psíquicos, involucra la perspectiva de la personalidad, el comportamiento del individuo y su relación social con las formas de organización del trabajo ^(^ ^16^ ^)^ . 

 La presencia de adicción al trabajo y agotamiento entre los profesionales de la salud puede estar relacionada con las características de la práctica médica, que no admite errores o fallos en la conducta asistencial. Con el fin de evitar errores en el proceso de cuidado, el enfermero tiende a trabajar arduamente y a estar sobrecargado de tareas ^(^ ^36^ ^-^ ^37^ ^)^ . 

 En este contexto, las personas adictas al trabajo tienen una fuerte motivación intrínseca para trabajar, a la cual no pueden resistirse. Esta motivación puede estar relacionada con la búsqueda de satisfacción y felicidad en el trabajo, mejora de la situación financiera, características del ambiente organizacional, presión impuesta por superiores, promoción en el empleo o incluso como alternativa de escape de los conflictos familiares ^(^ ^38^ ^)^ . 

 Es evidente que el exceso de trabajo, una de las dimensiones del *workaholism* , ocasiona daños al bienestar psicofísico y vocacional. El poco tiempo dedicado al descanso conduce a la exhaución cognitiva y emocional del trabajador, contribuyendo al bajo rendimiento ocupacional, interferencias en la interacción con otras personas, insatisfacción con el trabajo, fatiga crónica, comportamientos agresivos, irritabilidad, pensamientos negativos, frustración y desesperanza. Consecuentemente, hay un aumento del absentismo y la rotación entre estos profesionales ^(^ ^30^ ^,^ ^38^ ^)^ . 

 El agotamiento puede ser desencadenado por situaciones de exposición crónica a factores estresantes, siendo que el estrés ocupacional es otro factor que puede estar relacionado con los componentes del *workaholism* ^(^ ^39^ ^)^ . La enfermería es una categoría profesional que se caracteriza por asumir múltiples responsabilidades, lo que hace al enfermero propenso al trabajo excesivo, además de estar expuesto al riesgo de adicción al trabajo y a un ambiente que colabora para el desarrollo de estrés percibido y estrés traumático secundario ^(^ ^40^ ^-^ ^42^ ^)^ . 

 Reforzando los hallazgos sobre la relación entre *workaholism* y estrés ocupacional, es observable que los enfermeros con tendencias a la adicción al trabajo presentan mayores niveles de estrés traumático secundario ^(^ ^27^ ^,^ ^30^ ^,^ ^33^ ^-^ ^34^ ^)^ . Una explicación plausible para esta relación es que los trabajadores *workaholics* suelen presentar características de perfeccionismo. Las personas perfeccionistas tienen mayor dificultad para delegar tareas, creyendo que son los únicos capaces de realizar una determinada actividad. Estas actitudes se reflejan en el involucramiento en conflictos con los colegas de trabajo y ocasiona tensiones en sus relaciones interpersonales. Como consecuencia de estas interacciones negativas, se identifican niveles más altos de estrés ^(^ ^2^ ^)^ . 

 Cuanto mayor es el tiempo de exposición del enfermero a eventos negativos en el trabajo, mayor es la probabilidad de efectos negativos en otras áreas de su vida personal. Las pocas horas que quedan en el día a día de los enfermeros *workaholics* son insuficientes para que puedan adoptar estrategias para reducir el estrés y protegerse contra el agotamiento ^(^ ^43^ ^-^ ^44^ ^)^ . 

 Otro punto que requiere atención es que la constante dedicación al trabajo, sin considerar la necesidad de descanso del cuerpo y de la mente, puede llevar al estrés crónico que, a su vez, puede ocasionar problemas de salud más graves, como trastornos mentales, problemas relacionados con el sueño, consumo excesivo de Sustancias Psicoactivas (SPAs), problemas cognitivos, enfermedades endocrinas y afecciones cardiovasculares ^(^ ^45^ ^)^ . 

 Personas con estas características tienen dificultades para lidiar con el estrés ocupacional y pueden desarrollar problemas de salud mental ^(^ ^47^ ^)^ . Se cree que, ante situaciones amenazantes, el trabajo puede actuar como un mecanismo de escape relacionado con sentimientos negativos. Otra explicación para esta relación puede ser que las personas ansiosas temen fallar y/o rechazar tareas recibidas, mientras que las personas deprimidas trabajan más lentamente y necesitan compensar trabajando más horas para completar el trabajo. Ambas actitudes resultan en trabajo y carga horaria excesiva ^(^ ^48^ ^)^ . 

 Además, al considerar que los trabajadores caracterizados como *workaholics* son personas que piensan de forma persistente y frecuente en las demandas del trabajo, suelen evitar, muchas veces, la interacción social. Al adoptar estos hábitos, el trabajador se vuelve vulnerable a manifestar síntomas de humor negativo y depresión, incluso cuando no están involucrados con las actividades laborales ^(^ ^15^ ^,^ ^49^ ^)^ . 

 Además de la depresión, el sentimiento de culpa y la ansiedad son comúnmente experimentados por los *workaholics* , incluso, uno de los ítems de la escala de medición de la adicción al trabajo afirma: “Me siento culpable cuando tomo un descanso del trabajo” ^(^ ^50^ ^)^ . Los síntomas de ansiedad se sienten porque los *workaholics* , generalmente, están orientados a alcanzar sus objetivos de forma competitiva. Por lo tanto, el tiempo dedicado a actividades no relacionadas con el trabajo puede verse como un período durante el cual están “impedidos” de competir ^(^ ^51^ ^)^ . 

 La presencia de estos síntomas característicos de sufrimiento mental y el propio *workaholism* contribuyen a que puedan desarrollarse problemas relacionados con el sueño. Los trabajadores con adicción al trabajo presentan un mayor período de latencia del sueño, ya que el ritmo de trabajo excesivo es uno de los factores que afecta las etapas del sueño ^(^ ^31^ ^,^ ^52^ ^)^ . Los trabajadores clasificados como *workaholics* pueden aún presentar quejas de sueño insuficiente, dificultad para despertarse por la mañana (DAM), somnolencia excesiva diurna (SED), sensación de cansancio al despertar e insomnio ^(^ ^15^ ^,^ ^26^ ^)^ . 

 La dificultad y el cansancio al despertar por la mañana tienen una mayor asociación con el componente cognitivo del *workaholism* . En relación a este componente, el bajo distanciamiento psicológico del trabajo predice activación matinal negativa y fatiga. El pensar de forma persistente y frecuente en el trabajo, incluso cuando no se está trabajando, puede causar excitación autonómica y sufrimiento emocional por medio de la activación cognitiva, lo que resulta en mayor fatiga, similar a lo que ocurre en el insomnio ^(^ ^53^ ^-^ ^54^ ^)^ . 

 Estos déficits en la calidad del sueño pueden repercutir en el desempeño cognitivo y físico, en la disminución del estado de alerta, el humor, la atención y la memoria y puede estar asociado a un aumento del riesgo de errores y accidentes en el trabajo ^(^ ^55^ ^)^ . Además, las altas demandas de tareas ocasionan tensión mental y física, lo que puede resultar en siestas involuntarias y potencializar los riesgos de errores en la asistencia al paciente y posibles accidentes laborales ^(^ ^29^ ^)^ . 

 Otro escenario de actuación del enfermero, investigado por los estudios, fue el área de la docencia en la enseñanza superior. Entre enfermeros docentes, trabajar de forma compulsiva, excesiva y ser *workaholic* aumenta las chances de altos niveles de agotamiento emocional, despersonalización y baja eficacia profesional, dimensiones interdependientes del *burnout* ^(^ ^32^ ^)^ . 

 Las funciones del docente en la enseñanza superior están relacionadas con las múltiples actividades que se suman al tiempo restringido para su ejecución, lo que hace que el trabajo sea agotador. Además, se identifica que el profesor tiene pensamientos frecuentes y persistentes en el trabajo, lo que caracteriza ese trabajo también como compulsivo, pues hay una constante exigencia por productividad alineada a la competitividad del ambiente académico ^(^ ^56^ ^)^ . 

 Además del agotamiento, la ansiedad laboral entre los enfermeros docentes también fue uno de los factores asociados a las dimensiones del *workaholism* ^(^ ^31^ ^)^ . El ritmo intenso de trabajo hace que el profesor se acostumbre y no pueda desvincularse mentalmente de las actividades laborales. Este comportamiento suele relacionarse con sentimientos de culpa en los momentos de descanso y/o vacaciones, incluso si este profesional percibe que el trabajo lo está afectando de manera negativa ^(^ ^57^ ^)^ . 

 Los estudios incluidos presentaron, en el método, el diseño transversal, clasificados con nivel de evidencia VI ^(^ ^24^ ^)^ . El tipo de estudio adoptado converge con los objetivos planteados en las investigaciones, ya que permite la observación de las variables de interés en un momento determinado y de forma directa por el investigador, siendo particularmente útil para estudiar la prevalencia de un fenómeno en una población determinada. Además, los estudios transversales buscan analizar las relaciones entre factores de riesgo, factores determinantes y lo que se suponen son sus consecuencias o efectos ^(^ ^58^ ^)^ . 

 A pesar del bajo nivel de evidencia, los investigadores pueden utilizar herramientas para mejorar la calidad de la presentación de los resultados de estudios transversales, como la iniciativa *Strengthening the Reporting of Observational studies in Epidemiology* (STROBE) ^(^ ^59^ ^)^ . Al analizar los estudios, se observó que la mayoría buscó seguir cuidadosamente estas recomendaciones como forma de garantizar la calidad metodológica de la investigación, reflejada mediante la puntuación obtenida con la aplicación del *Checklist for Analytical Cross-Sectional Studies* ^(^ ^25^ ^)^ . 

 En cuanto a las limitaciones, se señala el nivel de evidencia de las producciones incluidas, ya que, al ser estudios transversales, no es posible hacer inferencias acerca de la relación causa y efecto entre *workaholism* y la salud mental de los enfermeros. Se señala que algunos estudios incluidos presentaban debilidades en la descripción metodológica, como la no especificación del sector de actuación del enfermero, la descripción insuficiente sobre la recolección de datos y la negligencia de factores de confusión. A pesar de las limitaciones identificadas, se buscó seguir cuidadosamente las recomendaciones para el desarrollo de revisiones integrativas. 

## Conclusión

 Las evidencias científicas demostraron que el *workaholism* estuvo relacionado con el estrés percibido en el trabajo, agotamiento emocional, despersonalización y síntomas ansiosos y depresivos, lo que resultó en baja eficacia profesional y mala calidad del sueño entre los profesionales adictos al trabajo. 

 El *workaholism* no debe categorizarse solo como un problema de índole psicológico e individual, sino también como un problema social que influye en las formas de organización del trabajo, en el desempeño de las actividades técnico-científicas de los enfermeros y en las relaciones terapéuticas con el cliente, ya que contribuye al padecimiento mental del trabajador, el absentismo y las licencias laborales. 

 Se sugiere que se realicen nuevas investigaciones sobre las repercusiones del *workaholism* en la salud mental de los enfermeros en escenarios de actuación, como en la Atención Primaria de Salud, así como estudios que evalúen, de manera rigurosa, la relación causa y efecto entre *workaholism* y síntomas psicoemocionales, teniendo en cuenta que al ser de abordaje transversal, los estudios incluidos no permiten establecer una relación temporal entre la exposición y el efecto, lo que dificulta determinar una relación causal. 

 Los resultados de esta revisión integrativa podrán contribuir a profundizar el conocimiento sobre los factores asociados al *workaholism* en enfermeros, abordando la influencia de las condiciones de trabajo sobre la salud ocupacional y mental, además de proporcionar reflexiones acerca de la prevención de comportamientos adictivos en el trabajo e incentivar la implementación de políticas públicas para la promoción de la salud del trabajador enfocadas en el enfermero y demás miembros del equipo de enfermería. 

# Translation: Fatores associados ao *workaholism* na saúde mental de enfermeiros: revisão integrativa

**DOI:** 10.1590/1518-8345.7046.4219

**Authors:** Nanielle Silva Barbosa, Jefferson Abraão Caetano Lira, Amanda Alves de Alencar Ribeiro, Eukália Pereira da Rocha, Maria José Quina Galdino, Márcia Astrês Fernandes

**Affiliations:** 1 Universidade Federal do Piauí, Departamento de Pós-Graduação em Enfermagem, Teresina, PI, Brasil.; 2 Bolsista da Coordenação de Aperfeiçoamento de Pessoal de Nível Superior (CAPES), Brasil.; 3 Universidade Estadual do Norte do Paraná, Departamento de Enfermagem, Bandeirantes, PR, Brasil.; 4 Bolsista do Conselho Nacional de Desenvolvimento Científico e Tecnológico (CNPq), Brasil.

**Keywords:** Enfermeiros e Enfermeiras, Comportamento Aditivo, Trabalho, Condições de Trabalho, Saúde Mental, Saúde Ocupacional

## Abstract

**Objetivo:**

sintetizar as principais evidências científicas disponíveis sobre os fatores associados ao *workaholism* na saúde mental de enfermeiros.

**Método:**

revisão integrativa realizada em sete bases de dados. A amostra foi composta por 11 estudos. A classificação do Nível de Evidência seguiu o modelo descrito por Melnyk e Fineout-Overholt. A qualidade metodológica foi avaliada por meio do *Checklist for Analytical Cross-Sectional Studies* . A análise e síntese dos dados foram realizadas de maneira qualitativa e descritiva, respectivamente.

**Resultados:**

os fatores associados ao *workaholism* foram *burnout* , estresse, ansiedade, depressão, problemas relacionados ao sono, baixa capacidade de concentração e incidentes negativos no trabalho, os quais afetaram a saúde mental dos enfermeiros.

**Conclusão:**

a síntese revelou que o *workaholism* apresentou relação com o estresse percebido no trabalho, exaustão emocional, despersonalização e sintomas ansiosos e depressivos, o que resultou em baixa eficácia profissional e má qualidade no sono entre os profissionais viciados no trabalho.

## Destaques

**(1)** Estresse e esgotamento profissional foram relacionados ao *workaholism* .

**(2)** Sintomas de ansiedade e depressão podem ser desencadeados devido ao *workaholism* .

**(3)** O *workaholism* contribui para problemas de sono e incidentes negativos no trabalho.

**(4)** *Workaholism* interfere na qualidade da assistência de enfermagem.

## Introdução

 O *workaholism* é uma expressão americana utilizada para designar o vício em trabalho, também conhecida como adição ao trabalho, ela faz referência à dependência psicopatológica do indivíduo em relação às suas atividades de trabalho, estando entre as principais causas de adoecimento físico e mental em trabalhadores. Esse fenômeno se desenvolve progressivamente e se manifesta por meio de condutas laborais que atingem aspectos diversos, principalmente os relacionados às demandas social, ocupacional e de saúde ^(^ ^1^ ^)^ . 

 Na caracterização do estado de adição ao trabalho, duas importantes dimensões de avaliação são delimitadas: trabalho excessivo e compulsivo. O trabalho excessivo está associado à dimensão comportamental e refere-se à conduta aditiva de trabalhar excessivamente por um período extenso de horas, além de apresentar dificuldade em se desvincular do trabalho em momentos de descanso ou férias e envolver-se em múltiplos projetos simultâneos. O trabalho compulsivo está relacionado à dimensão cognitiva e associa-se à pressão interna cognitiva, crenças e pensamentos correlacionados ao trabalho que levam aos comportamentos e atitudes de compulsão ^(^ ^2^ ^)^ . 

 O *workaholism* também é associado ao prazer e à satisfação laboral. Entretanto, essa correlação vem sendo questionada e modificada em decorrência do adoecimento mental e de manifestações somáticas provocadas pelo vício em trabalho ^(^ ^3^ ^)^ . Outras nuances comportamentais relacionadas ao trabalho podem ser confundidas com o *workaholism* . O engajamento no trabalho, por exemplo, também se associa a cargas horárias excessivas e ao extenso envolvimento com as atividades laborais. No entanto, diferentemente da adição, o engajamento interliga-se a um desempenho satisfatório no trabalho em conjunto com sentimentos de empoderamento, afeto positivo e qualidade em saúde ^(^ ^4^ ^)^ . 

 Entende-se que as circunstâncias e as motivações associadas ao *workaholism* pertencem a uma complexidade de fatores multidimensionais, nos quais podem ser incluídos: oscilação ou falta de autoestima, sentimento de inferioridade, medo do fracasso, desejo de realização, exigências organizacionais acentuadas e pressão social, devido à valorização constante de alta produtividade e desempenho ^(^ ^5^ ^)^ . 

 Os *workaholics* ou “viciados em trabalho”, ao desempenharem suas funções de forma excessiva e compulsiva, abdicam de momentos de descanso, lazer e/ou de interações sociais com cônjuges, familiares e amigos. Todavia, apesar da intensa dedicação ao trabalho, geralmente não conseguem atingir a performance desejada em decorrência do aumento da vulnerabilidade à incapacidade laborativa e dos impactos na saúde biopsicossocial provocados pelo *workaholism* ^(^ ^6^ ^)^ . 

 Apesar desse fenômeno apresentar ampla prevalência em categorias profissionais diversas, como 42,1% ^(^ ^7^ ^)^ entre engenheiros, 44,9% ^(^ ^8^ ^)^ entre médicos e 58,3% ^(^ ^9^ ^)^ entre treinadores esportivos, nota-se o tropismo acentuado entre *workaholism* e enfermeiros ^(^ ^10^ ^-^ ^11^ ^)^ . Esses trabalhadores estão expostos a situações e eventos que podem levar a comportamentos disfuncionais e ampliar os riscos à dependência no trabalho. 

 Enfermeiros lidam diariamente com demandas que envolvem a proximidade com pacientes e familiares, exigindo um domínio de habilidades interpessoais, empatia e compaixão. As exigências pelo desenvolvimento efetivo dessas competências em conjunto com a elevada carga e demanda de trabalho (superior a 40 horas semanais) podem desencadear repercussões negativas à saúde desses profissionais, com o aumento do esgotamento psicológico ( *Burnout* ), estresse traumático secundário e *workaholism* ^(^ ^12^ ^)^ . 

 Comportamentos aditivos entre enfermeiros e seus fatores associados permitem um delineamento explicativo da dimensão dessa problemática. Tais indicativos e considerações revelam a expressiva relação entre o *workaholism* e as alterações no bem-estar físico e psicológico do enfermeiro ^(^ ^1^ ^,^ ^13^ ^-^ ^14^ ^)^ . 

 Estudos de prevalência estimam taxas médias entre 13,77% ^(^ ^15^ ^)^ e 37% ^(^ ^16^ ^)^ de enfermeiros *workaholics* . Aos enfermeiros *workaholics* , associam-se agravos de saúde físicos e mentais, como dificuldades para dormir e/ou manter-se acordado durante o trabalho, quadros de depressão leve a moderada e implicações negativas na interação social, familiar e na qualidade da assistência prestada aos pacientes ^(^ ^12^ ^,^ ^15^ ^,^ ^17^ ^)^ . 

 Nota-se que a adição ao trabalho se associa a fatores multidimensionais. As evidências acerca do *workaholism* entre os enfermeiros são importantes para nortear a promoção da saúde ocupacional, com intuito de melhorar a saúde mental, a qualidade de vida, a satisfação e o desempenho no trabalho e, por conseguinte, a qualidade da assistência de enfermagem prestada, além de identificar possíveis lacunas a serem investigadas sobre a temática. 

 A análise crítica e detalhada sobre os fatores associados ao *workaholism* é imprescindível para a ampliação do entendimento sobre os aspectos inerentes à adição ao trabalho. Além disso, essas evidências poderão contribuir para nortear os gestores em saúde no planejamento de estratégias preventivas direcionadas à construção de comportamentos protetores à saúde mental dos trabalhadores nos contextos laborais. 

 A partir do exposto, este estudo tem como objetivo sintetizar as principais evidências científicas disponíveis sobre os fatores associados ao *workaholism* na saúde mental de enfermeiros. 

## Métodos

### Tipo de estudo

 Trata-se de uma revisão integrativa da literatura, na qual foram percorridas as seguintes etapas: elaboração da questão norteadora, busca e seleção dos estudos primários, avaliação dos estudos primários, análise dos dados e apresentação da revisão ^(^ ^18^ ^)^ . A redação do estudo seguiu as recomendações do *Preferred Reporting Items for Systematic Reviews and Meta-Analyses* (PRISMA) ^(^ ^19^ ^)^ . 

 O protocolo desta revisão foi registrado na *Open Science Framework* (OSF) e encontra-se disponível para acesso por meio do link: https://osf.io/r9pnw/ , apresentando o identificador DOI: 10.17605/OSF.IO/R9PNW ^(^ ^20^ ^)^ . 

### Período

O estudo foi realizado no período de 01 de março a 31 de julho de 2023.

### Questão norteadora

 A questão norteadora definida para conduzir esta revisão integrativa foi: “quais as evidências científicas acerca dos fatores associados ao *workaholism* na saúde mental de enfermeiros?”. Para a elaboração dessa questão, foi adotado o acrônimo PICo (População, Interesse e Contexto) ^(^ ^21^ ^)^ , sendo P = população (enfermeiros), I = interesse (fatores associados ao *workaholism* na saúde mental) e o Co = contexto (trabalho). 

### Critérios de elegibilidade

Foram considerados critérios de inclusão: estudos primários relacionados à temática, realizados com enfermeiros, sem delimitação temporal e de idioma. Os critérios de exclusão foram: trabalhos de conclusão de curso, dissertações, teses, editoriais e aqueles que não respondessem à questão norteadora.

### Busca e seleção dos estudos

 A busca dos estudos primários ocorreu em 05 de abril de 2023, nas bases de dados *Medical Literature Analysis and Retrieval System on-line* (MEDLINE) via PubMed, *Cumulative Index to Nursing and Allied Health Literature* (CINAHL-Ebsco), *Web of Science Core Collection* , Scopus, Embase, Base de Dados em Enfermagem (BDENF) e Literatura Latino-Americana e do Caribe em Ciências da Saúde (LILACS), via Biblioteca Virtual em Saúde (BVS). As bases de dados foram acessadas gratuitamente por meio do Portal de Periódicos da Coordenação de Aperfeiçoamento de Pessoal de Nível Superior (CAPES). A seleção dos estudos primários foi realizada entre 06 de abril e 20 de maio de 2023. 

Inicialmente, realizou-se um levantamento prévio sobre o tema, com a finalidade de identificar os principais termos em português e inglês utilizados como descritores e palavras-chave nos estudos. Em seguida, os descritores e palavras-chave foram estabelecidos, conforme o acrônimo PICo, de acordo com as especificidades das bases de dados.

 Os termos de busca selecionados no *Medical Subject Headings* (MeSH) foram aplicados na MEDLINE, *Web of Science Core Collection* e *Scopus* , os CINAHL *Subject* *Headings* na CINAHL e os *Emtree Terms* na *Embase* . Os termos em português, espanhol e inglês, selecionados nos Descritores em Ciências da Saúde (DeCS), foram utilizados nas bases de dados BDENF e LILACS. As estratégias de busca foram elaboradas por meio da combinação de descritores e palavras-chave, a partir dos operadores booleanos *OR* e *AND* , de acordo com a Figura 1 . 

**Figura 1:** - Estratégias de busca nas bases de dados consultadas para realização da revisão integrativa. Teresina, PI, Brasil, 2023

**Base de dados**	**Estratégias de busca**
MEDLINE, via PubMed	((((“nurses”[MeSH Terms]) OR (“nurse”[All Fields])) OR (“nursing personnel”[All Fields])) AND (((“workaholism”[All Fields]) OR (“workaholic”[All Fields])) OR (“work addiction”[All Fields]))) AND ((“work”[MeSH Terms]) OR (“work”[All Fields]))
CINAHL	( (MH “Nurses”) OR “Nurses” OR “nurse” OR “‘’Nursing Personnel’’” ) AND ( “workaholism” OR “workaholic” OR “‘’work addiction’’” ) AND ( (MH “Work”) OR “work” )
*Web of Science Core Collection*	(ALL=(nurses) OR ALL=(nurse) OR ALL=(‘’nursing personnel’‘)) AND (ALL=(workaholism ) OR ALL=(workaholic ) OR ALL=(’‘work addiction’’)) AND (ALL=(work))
Scopus	(( TITLE-ABS-KEY ( nurse ) OR TITLE-ABS-KEY ( nurses ) OR TITLE-ABS-KEY ( ‘’nursing personnel’’ ) )) AND (( TITLE-ABS-KEY ( workaholism ) OR TITLE-ABS-KEY ( workaholic ) OR TITLE-ABS-KEY ( ‘’work addiction’’ ) )) AND (TITLE-ABS-KEY ( work ))
Embase	(‘nurse’/exp OR nurse OR ‘community health nurse’ OR ‘community health nurses’ OR ‘nurse’ OR ‘nurse, community health’ OR ‘nurses’ OR ‘nurses, community health’ OR ‘nurses, public health’ OR ‘public health nurse’ OR ‘public health nurses’) AND (‘workaholism’/exp OR workaholism OR ‘work addiction’ OR ‘workaholic behavior’ OR ‘workaholism’) AND (‘work’/exp OR work OR ‘job’ OR ‘job description’ OR ‘work’)
BDENF e LILACS, via BVS	((mh:(Enfermeiras e Enfermeiros)) OR (Enfermeira) OR (Enfermeira e Enfermeiro) OR (Enfermeiras) OR (Enfermeiro e Enfermeira) OR (Enfermeiros e Enfermeiras)) AND ((mh:(‘’Comportamento aditivo’‘)) OR (’‘Conduta aditiva’’)) AND ((mh:(Trabalho)) OR (Trabalho)) AND ( db:(“LILACS” OR “BDENF”)) ((mh:(“Enfermeras y Enfermeros”)) OR (Enfermeras ) OR (Enfermeros)) AND ((mh:(“Conducta Adictiva”)) OR (“Conducta Adictiva”)) AND ((mh:(Trabajo)) OR (Trabajo)) AND ( db:(“LILACS” OR “BDENF”)) ((mh:(nurses)) OR (nurses)) AND ((mh:(“Behavior, Addictive”)) OR (“Behavior, Addictive”)) AND ((mh:(work)) OR (work)) AND ( db:(“LILACS” OR “BDENF”))

Destaca-se que os descritores e as palavras-chave referentes à Saúde Mental e ao Transtorno Mental, bem como seus correspondentes em inglês, utilizados para atender ao fenômeno de interesse, limitaram as buscas. Logo, não se obteve retorno de publicações nas bases de dados. Em decorrência disso, esses termos não constituíram elementos das estratégias de busca.

 Os resultados identificados nas bases de dados foram exportados para o *software online* *Rayyan* ^(^ ^22^ ^)^ , que auxiliou na detecção e exclusão de duplicatas e na seleção dos estudos incluídos na revisão. A seleção dos estudos foi realizada por dois revisores, de forma independente, em duas etapas e seguiu as recomendações (identificação, triagem e inclusão) do fluxograma PRISMA ^(^ ^19^ ^)^ . Dessa forma, na primeira etapa, houve a leitura dos títulos e resumos e a aplicação dos critérios de elegibilidade. Posteriormente, os revisores se reuniram para discutir as disparidades na seleção e chegarem a um consenso. Na etapa seguinte, realizou-se a leitura dos textos na íntegra e foram aplicados, novamente, os critérios de elegibilidade. Situações de discordância, ao final da etapa, foram resolvidas com o parecer de um terceiro revisor. 

Ressalta-se que a busca manual na lista de referências dos estudos primários incluídos foi realizada com a finalidade de identificar evidências adicionais relacionadas à temática de interesse.

## Coleta de dados

 A coleta dos dados correspondente à caracterização dos estudos ocorreu mediante a utilização de um formulário de extração de dados adaptado ^(^ ^23^ ^)^ , sendo extraídas as seguintes variáveis: título, autoria, ano de publicação, país, periódico, objetivo do estudo, desenho do estudo, principais resultados, nível de evidência e qualidade metodológica. 

A referida etapa foi realizada por dois revisores, de forma independente, no mês de maio de 2023. Nos casos em que ocorreram divergências, foi realizada reunião para discussão até que ocorresse um consenso.

### Tratamento e análise de dados

 A análise e síntese dos dados foram realizadas de maneira qualitativa e descritiva, respectivamente. Para a classificação do nível de evidência (NE) dos estudos foi utilizado modelo proposto por Melnyk e Fineout-Overholt ^(^ ^24^ ^)^ o qual é dividido nos seguintes níveis: nível I – evidências oriundas de revisão sistemática ou metanálise de todos ensaios clínicos relevantes randomizados controlados ou provenientes de diretrizes clínicas baseadas em revisões sistemáticas de ensaios clínicos randomizados controlados; nível II – evidências derivadas de, pelo menos, um ensaio clínico randomizado controlado bem delineado; nível III – evidências obtidas de ensaios clínicos bem delineados sem randomização; nível IV – evidências provenientes de estudos de coorte e de caso-controle bem delineados; nível V – evidências originárias de revisão sistemática de estudos descritivos e qualitativos; nível VI – evidências derivadas de um único estudo descritivo ou qualitativo; e nível VII – evidências oriundas de opinião de autoridades e/ou relatórios de comitês de especialistas. O *Checklist for Analytical Cross-Sectional Studies* foi utilizado para avaliar a qualidade metodológica das publicações. Essa ferramenta é composta por oito perguntas. Não há um escore mínimo para determinar se uma pesquisa possui qualidade ou não, porém, quanto mais respostas “sim (S)”, melhor a qualidade metodológica identificada ^(^ ^25^ ^)^ . 

As informações obtidas foram apresentadas por meio de quadros, nos quais os estudos encontram-se caracterizados conforme as variáveis de interesse coletadas.

## Resultados

 A busca nas bases de dados retornou 1026 publicações, sendo que 177 foram removidas por duplicatas. Desse modo, 849 estudos foram submetidos à leitura do título e do resumo. Após aplicação dos critérios de elegibilidade, 825 estudos foram excluídos por não responderem à questão norteadora e sete estudos por envolverem outras categorias profissionais, restando 17 estudos que foram lidos na íntegra. Após essa etapa, cinco estudos foram excluídos por não responderem à questão norteadora, dois estudos por serem revisões de literatura e dois pelo fato de serem protocolos. Assim sendo, oito estudos primários foram selecionados para compor a amostra. A leitura da lista de referência permitiu a inclusão de mais três artigos, totalizando 11 estudos. Na Figura 2 , encontra-se o fluxograma da etapa de seleção dos estudos primários incluídos nesta revisão. 

**Figura 2:** - Fluxograma de seleção dos estudos incluídos na revisão integrativa. Teresina, PI, Brasil, 2023

 A síntese descritiva dos estudos primários conforme autoria, ano de publicação, país do estudo, periódico, tipo de estudo, amostra, cenário e nível de evidência é apresentada na Figura 3 . 

**Figura 3:** - Síntese dos estudos incluídos na revisão integrativa. Teresina, PI, Brasil, 2023

**Autoria e ano**	**País**	**Periódico**	**Tipo de estudo, amostra e cenário**	**NE** ^*^
Ariapooran (2019) ^(^ ^15^ ^)^	Irã	*Iranian Journal of Nursing and Midwifery Research*	Estudo analítico transversal e correlacional Amostra: n= 247 Cenário: três hospitais Setor de atuação: não especificado	VI
Adolfo, et al. (2021) ^(^ ^16^ ^)^	Arábia Saudita	*Perspective in Psychiatric Care*	Estudo transversal Amostra: n= 427 Cenário: quatro hospitais públicos terciários Setor de atuação: emergência, ambulatório, clínica médica, clínica cirúrgica, nefrologia, centro obstétrico, unidade de terapia intensiva e administração	VI
Kubota, et al. (2010) ^(^ ^26^ ^)^	Japão	*Industrial Health*	Estudo transversal Amostra: n= 312 Cenário: dois hospitais universitários Setor de atuação: clínica médica, clínica cirúrgica e emergência	VI
Jenaabadi, et al. (2017) ^(^ ^27^ ^)^	Irã	*Journal of Health Promotion Management*	Estudo descritivo e correlacional Amostra: n= 350 Cenário: hospitais universitários Setor de atuação: não especificado	VI
Nonnis, et al. (2018) ^(^ ^28^ ^)^	Itália	*Open Psychology Journal*	Estudo transversal Amostra: n= 614 Cenário: seis hospitais ediatria (115, 18.7%), Oncologia (93, 15.1%), Em geral Medicamento (89, 14.4%), Psiquiatria (51, 8,3%), Obstetrícia (34, 5,5%), Neurologia (33, 5,3%), Emergência (32, 5,2%) e Radioterapia (28 ediatria (115, 18.7%), Oncologia (93, 15.1%), Em geral Medicamento (89, 14.4%), Psiquiatria (51, 8,3%), Obstetrícia (34, 5,5%), Neurologia (33, 5,3%), Emergência (32, 5,2%) e Radioterapia (28 ediatria (115, 18.7%), Oncologia (93, 15.1%), Em geral Medicamento (89, 14.4%), Psiquiatria (51, 8,3%), Obstetrícia (34, 5,5%), Neurologia (33, 5,3%), Emergência (32, 5,2%) e Radioterapia (28 Setor de atuação: clínica geral, pediatria, oncologia, psiquiatria, obstetrícia, neurologia, radioterapia e emergência	VI
Andreassen, et al. (2018) ^(^ ^29^ ^)^	Noruega	*Industrial Health*	Estudo transversal Amostra: n= 1781 Cenário: *online* Setor de atuação: não especificado	VI
Kwak, et al. (2018) ^(^ ^30^ ^)^	Coreia	*Journal of Addictions Nursing*	Estudo transversal Amostra: n= 278 Cenário: três hospitais universitários Setor de atuação: unidade de terapia intensiva, centro cirúrgico e emergência	VI
Almeida, et al. (2020) ^(^ ^31^ ^)^	Brasil	Revista Latino-Americana de Enfermagem	Estudo transversal Amostra: n= 333 Cenário: 47 universidades públicas brasileiras Setor de atuação: Programas de Pós-Graduação em Enfermagem	VI
Galdino, et al. (2021) ^(^ ^32^ ^)^	Brasil	Acta Paulista de Enfermagem	Estudo transversal Amostra: n= 368 Cenário: 47 universidades públicas brasileiras Setor de atuação: Programas de Pós-Graduação em Enfermagem	VI
Borges, et al. (2021) ^(^ ^33^ ^)^	Portugal e Espanha	*Journal of Nursing Management*	Estudo multicêntrico, comparativo e transversal Amostra: n= 333 Cenário: ambiente hospitalar Setor de atuação: não especificado	VI
Ruíz-Garcia, et al. (2022) ^(^ ^34^ ^)^	Espanha	*Intensive & Critical Care Nursing*	Estudo transversal, descritivo, quantitativo e correlacional Amostra: n= 219 Cenário: ambiente hospitalar Setor de atuação: unidade de terapia intensiva, unidade de cuidados intensivos cardíacos e emergência	VI

* NE = Nível de evidência

 Os anos de 2018 e 2021, juntos, concentraram seis produções ^(^ ^16^ ^,^ ^28^ ^-^ ^30^ ^,^ ^32^ ^-^ ^33^ ^)^ . Os estudos foram desenvolvidos em países da Ásia ^(^ ^15^ ^-^ ^16^ ^,^ ^26^ ^-^ ^27^ ^,^ ^30^ ^)^ , Europa ^(^ ^28^ ^-^ ^29^ ^,^ ^33^ ^-^ ^34^ ^)^ e América do Sul ^(^ ^31^ ^-^ ^32^ ^)^ , dos quais dois estão publicados no periódico *Industrial Health* ^(^ ^26^ ^,^ ^29^ ^)^ *.* Em relação à abordagem metodológica, os estudos caracterizaram-se como transversais ^(^ ^15^ ^-^ ^16^ ^,^ ^26^ ^-^ ^34^ ^)^ , com amostra de participantes entre 1781 ^(^ ^29^ ^)^ e 219 ^(^ ^34^ ^)^ enfermeiros, sendo realizados em cenário hospitalar ^(^ ^15^ ^-^ ^16^ ^,^ ^26^ ^-^ ^28^ ^,^ ^30^ ^,^ ^33^ ^-^ ^34^ ^)^ , *online* ^(^ ^29^ ^)^ e em universidades ^(^ ^31^ ^-^ ^32^ ^)^ e classificados com nível de evidência VI ^(^ ^15^ ^-^ ^16^ ^,^ ^26^ ^-^ ^34^ ^)^ . A Figura 4 traz os objetivos e as principais informações acerca dos resultados dos estudos incluídos. 

**Figura 4:** - Síntese dos estudos incluídos na revisão integrativa segundo objetivos e principais resultados. Teresina, PI, Brasil, 2023

**Estudo primário**	**Objetivo**	**Principais resultados**
Ariapooran (2019) ^(^ ^15^ ^)^	Investigar o papel do vício em trabalho na previsão de problemas de sono e depressão entre enfermeiros iranianos.	O *workaholism* foi positivamente correlacionado a problemas associados ao sono e depressão.
Adolfo, et al. (2021) ^(^ ^16^ ^)^	Determinar se as tendências *workaholic* e as variáveis demográficas dos enfermeiros predizem a qualidade de vida profissional.	Tendências *workaholic* foram fatores preditores de *burnout* e Estresse Traumático Secundário.
Kubota, et al. (2010) ^(^ ^26^ ^)^	Examinar a associação entre *workaholism* , tendência a trabalhar excessivamente de forma compulsiva e problemas de sono entre enfermeiros japoneses.	Foram identificadas associações entre o vício em trabalho e problemas relacionados à qualidade do sono.
Jenaabadi, et al. (2017) ^(^ ^27^ ^)^	Determinar a correlação do vício em trabalho com o estresse no trabalho e o esgotamento profissional em enfermeiros.	Houve uma correlação significativa e positiva entre *workaholism* e estresse relacionado ao trabalho e esgotamento profissional.
Nonnis, et al. (2018) ^(^ ^28^ ^)^	Contribuir para a pesquisa em andamento sobre a relação entre *workaholism* e *burnout* entre enfermeiros.	*O workaholism* foi apontado como preditor de *burnout* .
Andreassen, et al. (2018) ^(^ ^29^ ^)^	Investigar sobre condições de trabalho, sono e saúde.	O vício no trabalho foi identificado como preditor consistente de incidentes negativos relacionados ao trabalho, com destaque para cochilos involuntários no trabalho, o que quase ocasionou danos a pacientes e equipamentos.
Kwak, et al. (2018) ^(^ ^30^ ^)^	Delinear as relações entre a dependência do trabalho e qualidade de vida profissional entre enfermeiros em hospitais universitários.	O vício no trabalho foi apontado como fator influente de *burnout* e estresse traumático secundário.
Almeida, et al. (2020) ^(^ ^31^ ^)^	Identificar a prevalência e os fatores associados ao *workaholism* entre docentes de pós-graduação *stricto sensu* em enfermagem.	Fatores como sono insatisfatório, baixa capacidade de concentração e ansiedade laboral estiveram associados às dimensões do *workaholism.*
Galdino, et al. (2021) ^(^ ^32^ ^)^	Verificar a associação do *burnout* com *workaholism* e qualidade de vida entre docentes de mestrado e/ou doutorado em enfermagem.	Ser identificado como trabalhador *workaholic* aumentou significativamente as chances de altos níveis de exaustão emocional, despersonalização e baixa eficácia profissional.
Borges, et al. (2021) ^(^ ^33^ ^)^	Identificar e comparar os níveis de *workaholism* , *engagement* e interação familiar entre enfermeiros portugueses e espanhóis.	Enfermeiros com estresse pontuaram níveis médios mais altos para vício em trabalho e trabalho excessivo.
Ruíz-Garcia, et al. (2022) ^(^ ^34^ ^)^	Investigar a prevalência do vício em trabalho, bem como a relação entre interação trabalho-família em enfermeiros de emergência e cuidados intensivos.	Estresse percebido no trabalho foi relacionado ao *workaholism* .

 A respeito dos objetivos dos estudos, observou-se o interesse em examinar a associação entre *workaholism* e problemas relacionados ao sono ^(^ ^15^ ^,^ ^26^ ^)^ , delinear a relação entre *workaholism* , sintomas de ansiedade, depressão e qualidade de vida profissional ^(^ ^16^ ^,^ ^31^ ^)^ , correlacioná-lo com estresse e esgotamento profissional ^(^ ^27^ ^-^ ^28^ ^,^ ^30^ ^,^ ^32^ ^)^ , identificar a prevalência e os fatores associados ao *workaholism* e comparar os níveis de *workaholism* , engajamento e interação familiar ^(^ ^29^ ^,^ ^33^ ^-^ ^34^ ^)^ . Os principais resultados apontam que os fatores relacionados ao *workaholism* na saúde mental de enfermeiros foram depressão ^(^ ^15^ ^)^ , *burnout* ^(^ ^16^ ^,^ ^27^ ^-^ ^28^ ^,^ ^30^ ^,^ ^33^ ^-^ ^34^ ^)^ , estresse ^(^ ^16^ ^,^ ^27^ ^,^ ^30^ ^,^ ^33^ ^)^ , ansiedade ^(^ ^31^ ^)^ , problemas relacionados ao sono ^(^ ^15^ ^,^ ^26^ ^,^ ^29^ ^,^ ^31^ ^)^ , baixa capacidade de concentração ^(^ ^15^ ^,^ ^26^ ^,^ ^29^ ^,^ ^31^ ^)^ e incidentes negativos no trabalho ^(^ ^29^ ^)^ . 

 Na avaliação da qualidade metodológica, por meio do *Checklist for Analytical Cross-Sectional Studies* , duas pesquisas ^(^ ^27^ ^,^ ^29^ ^)^ receberam “sim” nos oito itens que compõem a ferramenta, uma pesquisa ^(^ ^28^ ^)^ recebeu “sim” em sete itens e oito estudos receberam “sim” em seis itens ^(^ ^15^ ^-^ ^16^ ^,^ ^26^ ^,^ ^30^ ^-^ ^34^ ^)^ , conforme apresentado na Figura 5 . 

**Figura 5:** - Qualidade metodológica dos estudos incluídos na revisão integrativa. Teresina, PI, Brasil, 2023

**Estudo primário**	**Q1**	**Q2**	**Q3**	**Q4**	**Q5**	**Q6**	**Q7**	**Q8**
Ariapooran (2019) ^(^ ^15^ ^)^	Sim	Sim	Sim	Sim	Não	Não	Sim	Sim
Adolfo, et al. (2021) ^(^ ^16^ ^)^	Sim	Sim	Sim	Sim	Não	Não	Sim	Sim
Kubota, et al. (2010) ^(^ ^26^ ^)^	Não	Sim	Sim	Sim	Sim	Não	Sim	Sim
Jenaabadi, et al. (2017) ^(^ ^27^ ^)^	Sim	Sim	Sim	Sim	Sim	Sim	Sim	Sim
Nonnis, et al. (2018) ^(^ ^28^ ^)^	Não	Sim	Sim	Sim	Sim	Sim	Sim	Sim
Andreassen, et al. (2018) ^(^ ^29^ ^)^	Sim	Sim	Sim	Sim	Sim	Sim	Sim	Sim
Kwak, et al. (2018) ^(^ ^30^ ^)^	Sim	Sim	Sim	Sim	Não	Não	Sim	Sim
Almeida, et al. (2020) ^(^ ^31^ ^)^	Sim	Sim	Sim	Sim	Não	Não	Sim	Sim
Galdino, et al. (2021) ^(^ ^32^ ^)^	Sim	Sim	Sim	Sim	Não	Não	Sim	Sim
Borges, et al. (2021) ^(^ ^33^ ^)^	Sim	Sim	Sim	Sim	Não	Não	Sim	Sim
Ruíz-Garcia, et al. (2022) ^(^ ^34^ ^)^	Sim	Sim	Sim	Sim	Não	Não	Sim	Sim

Q1= Os critérios de inclusão na amostra foram definidos de forma clara? Q2= Os sujeitos do estudo e cenário são descritos em detalhes? Q3= A exposição foi medida de forma válida e confiável? Q4= Foram utilizados critérios objetivos e padronizados para a medição da condição? Q5= Foram identificados fatores de confusão? Q6= Foram declaradas estratégias para lidar com fatores de confusão? Q7= Os resultados foram medidos de forma válida e confiável? Q8= Foi utilizada análise estatística apropriada?

## Discussão

 Os enfermeiros lidam de forma constante com demandas organizacionais e pessoais, de saúde física, mental e emocional e apresentam maior vulnerabilidade ao desenvolvimento de comportamentos aditivos associados ao trabalho, cujas consequências resultam em sofrimento psicológico e podem impactar diretamente na qualidade do trabalho desempenhado ^(^ ^1^ ^,^ ^35^ ^)^ . 

 Entre os estudos identificados, a maioria foi desenvolvida com enfermeiros atuantes no cenário hospitalar. Apontou-se que há relação entre o vício em trabalho e o esgotamento profissional ^(^ ^27^ ^-^ ^28^ ^,^ ^30^ ^)^ . Entretanto, estabelecer uma conexão entre ambos é algo complexo, pois essa relação é multifacetada, ou seja, além de fatores psíquicos, envolve a perspectiva da personalidade, do comportamento do indivíduo e da sua relação social com as formas de organização do trabalho ^(^ ^16^ ^)^ . 

 A presença do vício em trabalho e do esgotamento entre os profissionais de saúde pode ser relacionada às características da prática médica, que não admite erros ou falhas na conduta assistencial. Com o intuito de evitar erros no processo do cuidado, o enfermeiro tende a trabalhar arduamente e a estar sobrecarregado de tarefas ^(^ ^36^ ^-^ ^37^ ^)^ . 

 Nesse contexto, pessoas viciadas em trabalho têm uma forte motivação intrínseca para trabalhar, à qual não conseguem resistir. Essa motivação pode estar relacionada à busca por satisfação e felicidade no trabalho, melhora da situação financeira, características do ambiente organizacional, pressão imposta por superiores, promoção no emprego ou mesmo como alternativa de fuga dos conflitos familiares ^(^ ^38^ ^)^ . 

 É nítido que o excesso de trabalho, uma das dimensões do *workaholism* , ocasiona prejuízos ao bem-estar psicofísico e vocacional. O pouco tempo despendido para descanso leva à exaustão cognitiva e emocional do trabalhador, contribuindo para o baixo desempenho ocupacional, interferências na interação com outras pessoas, insatisfação com o trabalho, fadiga crônica, comportamentos agressivos, irritabilidade, pensamentos negativos, frustração e desesperança. Consequentemente, há o aumento do absenteísmo e rotatividade entre esses profissionais ^(^ ^30^ ^,^ ^38^ ^)^ . 

 O esgotamento pode ser desencadeado por situações de exposição crônica a fatores estressores, sendo que o estresse ocupacional é outro fator que pode ser relacionado aos componentes do *workaholism* ^(^ ^39^ ^)^ . A enfermagem é uma categoria profissional que se caracteriza por assumir múltiplas responsabilidades, o que torna o enfermeiro propenso ao trabalho excessivo, além de ser exposto ao risco do vício em trabalho e a um ambiente que colabora para o desenvolvimento de estresse percebido e estresse traumático secundário ^(^ ^40^ ^-^ ^42^ ^)^ . 

 Reforçando os achados sobre a relação entre *workaholism* e estresse ocupacional, é observável que os enfermeiros com tendências ao vício em trabalho apresentam maiores níveis de estresse traumático secundário ^(^ ^27^ ^,^ ^30^ ^,^ ^33^ ^-^ ^34^ ^)^ . Uma explicação plausível para essa relação é que os trabalhadores *workaholics* costumam apresentar características de perfeccionismo. Pessoas perfeccionistas possuem maior dificuldade em delegar tarefas, acreditando que são os únicos aptos a realizar determinada atividade. Essas atitudes refletem no envolvimento em conflitos com os colegas de trabalho e ocasiona tensões em suas relações interpessoais. Como consequência dessas interações negativas, níveis mais altos de estresse são identificados ^(^ ^2^ ^)^ . 

 Quanto maior o tempo de exposição do enfermeiro a eventos negativos no trabalho, maior a probabilidade de efeitos negativos em outras áreas da sua vida pessoal. As poucas horas que sobram no dia a dia dos enfermeiros *workaholics* são insuficientes para que consigam adotar estratégias para reduzir o estresse e se proteger contra o esgotamento ^(^ ^43^ ^-^ ^44^ ^)^ . 

 Outro ponto que exige atenção é que a constante dedicação ao trabalho, sem se considerar a necessidade de descanso do corpo e da mente pode levar ao estresse crônico que, por sua vez, pode ocasionar problemas de saúde mais graves, como transtornos mentais, problemas relacionados ao sono, consumo excessivo de Substâncias Psicoativas (SPAs), problemas cognitivos, doenças endócrinas e agravos cardiovasculares ^(^ ^45^ ^)^ . 

 Com relação ao sofrimento psíquico, proveniente do desenvolvimento de transtornos mentais e *workaholism,* evidencia-se que a presença de Transtorno Obsessivo-Compulsivo (TOC) e sintomas de ansiedade e/ou depressão podem levar ao vício em trabalho e vice-versa ^(^ ^1^ ^,^ ^15^ ^)^ . As pesquisas sugerem, inclusive, que os *workaholics* são mais ansiosos do que deprimidos ^(^ ^46^ ^)^ . 

 Pessoas com essas características possuem dificuldades em lidar com o estresse ocupacional e podem desenvolver problemas de saúde mental ^(^ ^47^ ^)^ . Acredita-se que, diante de situações ameaçadoras, o trabalho pode atuar como um mecanismo de fuga relacionado a sentimentos negativos. Outra explicação para essa relação pode ser que pessoas ansiosas temem falhar e/ou recusar tarefas recebidas, enquanto pessoas deprimidas trabalham mais devagar e precisam compensar trabalhando mais horas para concluir o trabalho. Ambas atitudes resultam em trabalho e carga horária excessiva ^(^ ^48^ ^)^ . 

 Ademais, ao considerar que os trabalhadores caracterizados como *workaholics* são pessoas que pensam de forma persistente e frequente nas demandas de trabalho, estes costumam evitar, muitas vezes, a interação social. Ao adotar esses hábitos, o trabalhador torna-se vulnerável a manifestar sintomas de humor negativo e de depressão, mesmo quando não estão envolvidos com as atividades laborais ^(^ ^15^ ^,^ ^49^ ^)^ . 

 Além da depressão, o sentimento de culpa e a ansiedade são comumente vivenciados por *workaholics* , inclusive, um dos itens da escala de mensuração do vício em trabalho afirma: “Sinto-me culpado quando tiro uma folga do trabalho” ^(^ ^50^ ^)^ . Os sintomas de ansiedade são sentidos porque os *workaholics* , geralmente, são orientados para atingir seus objetivos de forma competitiva. Portanto, o tempo dispendido em atividades não relacionadas ao trabalho pode ser visto como um período durante o qual eles são “impedidos” de competir ^(^ ^51^ ^)^ . 

 A presença desses sintomas característicos de sofrimento mental e o próprio *workaholism* contribuem para que problemas relacionados ao sono possam ser desenvolvidos. Os trabalhadores com vício em trabalho apresentam maior período de latência do sono, pois o ritmo de trabalho excessivo é um dos fatores que afeta os estágios de sono ^(^ ^31^ ^,^ ^52^ ^)^ . Trabalhadores classificados como *workaholics* podem, ainda, apresentar queixas de sono insuficiente, dificuldade para acordar pela manhã (DAM), sonolência excessiva diurna (SED), sensação de cansaço ao acordar e insônia ^(^ ^15^ ^,^ ^26^ ^)^ . 

 Dificuldade e cansaço ao despertar pela manhã possuem maior associação com o componente cognitivo do *workaholism* . Em relação a esse componente, o baixo distanciamento psicológico do trabalho prevê ativação matinal negativa e fadiga. O pensar de forma persistente e frequente no trabalho, mesmo quando não se está trabalhando, pode causar excitação autonômica e sofrimento emocional por meio da ativação cognitiva, o que resulta em maior fadiga, semelhante ao que ocorre na insônia ^(^ ^53^ ^-^ ^54^ ^)^ . 

 Tais déficits na qualidade do sono podem repercutir no desempenho cognitivo e físico, na diminuição do estado de alerta, do humor, atenção e memória e pode estar associado a um aumento do risco de erros e acidentes no trabalho ^(^ ^55^ ^)^ . Além do mais, as altas demandas de tarefas ocasionam tensão mental e física, o que pode resultar em cochilos involuntários e potencializar os riscos de erros na assistência ao paciente e possíveis acidentes laborais ^(^ ^29^ ^)^ . 

 Outro cenário de atuação do enfermeiro, investigado pelos estudos, foi a área da docência no ensino superior. Entre enfermeiros docentes, trabalhar de forma compulsiva, excessiva e ser *workaholic* aumenta as chances de altos níveis de exaustão emocional, despersonalização e baixa eficácia profissional, dimensões interdependentes do *burnout* ^(^ ^32^ ^)^ . 

 As funções do docente no ensino superior estão relacionadas às múltiplas atividades que se somam ao tempo restrito para sua execução, o que torna o trabalho exaustivo. Além disso, identifica-se que o professor possui pensamentos frequentes e persistentes no trabalho, o que caracteriza esse trabalho também como compulsivo, pois há uma constante exigência por produtividade alinhada à competitividade do ambiente acadêmico ^(^ ^56^ ^)^ . 

 Além do esgotamento, a ansiedade laboral entre enfermeiros docentes também foi um dos fatores associados às dimensões do *workaholism* ^(^ ^31^ ^)^ . O ritmo intenso de trabalho faz com que o professor se habitue e não consiga se desvincular mentalmente das atividades laborais. Esse comportamento costuma se relacionar a sentimentos de culpa nos momentos de folga e/ou descanso, mesmo que o profissional perceba que o trabalho o está afetando de forma negativa ^(^ ^57^ ^)^ . 

 Os estudos incluídos apresentaram, no método, o desenho transversal, classificados com nível de evidência VI ^(^ ^24^ ^)^ . O tipo de estudo adotado converge com os objetivos elencados nas investigações, uma vez que permite a observação das variáveis de interesse em um determinado momento e de forma direta pelo pesquisador, sendo particularmente útil para se estudar a prevalência de um fenômeno em determinada população. Além disso, os estudos transversais buscam analisar as relações entre fatores de risco, fatores determinantes e o que se supõe serem suas consequências ou efeitos ^(^ ^58^ ^)^ . 

 Apesar do baixo nível de evidência, os pesquisadores podem utilizar ferramentas para melhorar a qualidade da apresentação dos resultados de estudos transversais, como a iniciativa *Strengthening the Reporting of Observational studies in Epidemiology* (STROBE) ^(^ ^59^ ^)^ . Ao se analisar os estudos, foi observado que a maioria buscou seguir, criteriosamente, as recomendações como forma de garantir a qualidade metodológica da investigação, refletida por meio da pontuação obtida com a aplicação do *Checklist for Analytical Cross-Sectional Studies* ^(^ ^25^ ^)^ . 

 Com relação às limitações, pontua-se o nível de evidência das produções incluídas, uma vez que, por serem estudos transversais, não é possível fazer inferências acerca da relação causa-efeito entre *workaholism* e saúde mental de enfermeiros. Pontua-se que alguns estudos incluídos apresentavam fragilidades na descrição metodológica, como a não especificação do setor de atuação do enfermeiro, a descrição insuficiente acerca da coleta de dados e a negligência de fatores de confusão. Apesar das limitações identificadas, buscou-se seguir criteriosamente as recomendações para o desenvolvimento de revisões integrativas. 

## Conclusão

 As evidências científicas demonstraram que o *workaholism* apresentou relação com o estresse percebido no trabalho, exaustão emocional, despersonalização e sintomas ansiosos e depressivos, o que resultou em baixa eficácia profissional e má qualidade do sono entre os profissionais viciados no trabalho. 

 O *workaholism* não deve ser categorizado apenas como um problema de cunho psicológico e individual, mas também um problema social e que influencia nas formas de organização do trabalho, no desempenho das atividades técnico-científicas dos enfermeiros e nas relações terapêuticas com o cliente, pois contribui para o adoecimento mental do trabalhador, para o absenteísmo e para os afastamentos laborais. 

 Sugere-se que sejam realizadas novas investigações acerca das repercussões do *workaholism* tanto na saúde mental de enfermeiros em cenários de atuação, quanto na Atenção Primária à Saúde, bem como sugestiona-se o desenvolvimento de estudos que avaliem, de forma criteriosa, a relação causa e efeito entre *workaholism* e sintomas psicoemocionais, tendo em vista que, por serem de abordagem transversal, os estudos incluídos não permitem estabelecer uma relação temporal entre a exposição e o efeito, o que dificulta determinar uma relação causal. 

 Os resultados desta revisão integrativa poderão contribuir para aprofundar o conhecimento acerca dos fatores associados ao *workaholism* em enfermeiros, abordando a influência das condições de trabalho sobre a saúde ocupacional e mental, além de proporcionar reflexões a respeito da prevenção de comportamentos aditivos no trabalho e incentivar a implementação de políticas públicas para a promoção da saúde do trabalhador voltadas para o enfermeiro e demais membros da equipe de enfermagem.
